# Total Synthesis and Antimicrobial Evaluation of Pagoamide A

**DOI:** 10.3389/fchem.2021.741290

**Published:** 2021-09-14

**Authors:** Cheng-Han Wu, John Chu

**Affiliations:** Department of Chemistry, National Taiwan University, Taipei City, Taiwan

**Keywords:** pagoamide A, marine natural products, total synthesis, Hantzsch thiazole synthesis, solid-phase peptide synthesis, antimicrobial activity

## Abstract

Natural products are often the starting point for drug development and also the testing ground for synthetic methods. Herein we describe the total synthesis and antimicrobial evaluation of a marine natural product, pagoamide A, which is a macrocyclic depsipeptide with two backbone thiazole units and a dimethylated *N*-terminus. The two thiazole building blocks were synthesized from commercially available materials in four or fewer steps and employed directly in solid-phase peptide synthesis (SPPS) to afford pagoamide A. The use of SPPS ensured that the synthetic sequence is operationally straightforward and, if needed, permits modular substitution of building blocks to easily access diverse structural analogs. Our antimicrobial assays showed that pagoamide A has moderate activity against *Bacillus subtilis*.

## Introduction

Microbial natural products have historically been a fruitful source of bioactive small molecules (Newman and Cragg, 2020). Peptide natural products, including nonribosomal peptides (NRPs) (Fischbach and Walsh, 2006) and ribosomally-synthesized and post-translationally modified peptides (RiPPs) (Arnison et al., 2013), are highly represented in small molecule therapeutics in clinical use today, particularly for the treatment of microbial infections. Both are very attractive classes of natural products that have inspired scientists as they look for starting points for the development of new drugs. Once a natural product demonstrated its potential as a drug candidate, the ability to access it with high efficiency through chemical synthesis from simple starting materials, i.e., total synthesis, is critically important and play an integral role as it progresses through the drug development pipeline.

Peptide natural products like NRP and RiPP are biosynthesized modularly, the former by an enzymatic assembly line and the latter by the ribosome (Fischbach and Walsh, 2006; Arnison et al., 2013). In these two biosynthetic platforms, an amino acid building block is first activated at the expense of an ATP and then added to a growing peptide chain through an intermediary, i.e., the phosphopantetheine arm on the thiolation domain and tRNA for NRP and RiPP, respectively. Solid-phase peptide synthesis (SPPS) is a synthetic platform devised by chemists for the construction of consecutive amide bonds that operates in a similarly modular fashion (Coin et al., 2007). A variety of reagents have been developed for the activation of amino acids (Takayama et al., 2018) to ensure high efficiency in the formation of an amide bond on the solid support (resins). An excess of activated amino acid can be used to drive the reaction to near completion; meanwhile, workup and purification are reduced to simply washing the resins with the appropriate solvents. As such, SPPS has become the method of choice for the synthesis of linear peptides.

However, peptide natural products are much more complex than simple linearly connected strings of amino acids (Fischbach and Walsh, 2006; Arnison et al., 2013). Structural complexity poses a number of synthetic challenges that SPPS must meet before it can be applied routinely for the total synthesis of diverse peptide natural products (McIntosh et al., 2009). Our laboratory is interested in expanding the scope of SPPS beyond the mere formation of amide bonds. In particular, we see an opportunity to combine the versatility of conventional solution-phase organic chemistry and the high efficiency of SPPS into a multifaceted synthetic platform.

Here we report the total synthesis of pagoamide A (**1**) (Li et al., 2020), a natural product isolated from the culture of green algae *Derbesia* sp. Pagoamide A (**1**) is made of eight l- and three d-amino acids. It contains a number of structural features that prevents it from being synthesized by standard SPPS, including a dimethylated *N*-terminus, two thiazole moieties, and a depsipeptide macrocycle with 19 atoms. Matching spectral characterizations of **1** obtained by total synthesis and culture extracts confirmed its stereochemical assignment. We also evaluated its antimicrobial activities against a panel of common microbial pathogens.

## Materials and Methods

### Chemicals, Glassware, Microbes, and Growth Media

Wang resins were purchased from Novabiochem (Merck). Amino acid building blocks and coupling reagents were purchased from P3 BioSystems and Novabiochem (Merck). Reaction vessels for SPPS were custom made by Tung-Yin Glassware Co., Ltd., and other common glassware were purchased from the same vendor. Reagents and consumables for organic synthesis were purchased from Thermo Fisher Scientific Inc. and Sigma-Aldrich Inc. Growth media were prepared from premixed powders purchased from BioShop Canada Inc.

### Fmoc-Gly-NH_2_ (5)

HBTU (569 mg, 1.5 mmol) was added to a solution of Fmoc-Gly-OH (297 mg, 1.0 mmol) and DIPEA (348 μl, 2.0 mmol) in THF (5 ml) and stirred at room temperature for 10 min. Ammonium bicarbonate (79 mg, 1.0 mmol) was added slowly, and the mixture was stirred for another 8 h before THF was removed *in vacuo*. The remaining residue was dissolved in EtOAc, washed successively with 1N HCl and 1M NaOH, dried over anhydrous sodium sulfate (Na_2_SO_4_), concentrated under reduced pressure, and purified by silica gel column chromatography (*R*_*f*_ = 0.24, CH_2_Cl_2_/MeOH = 95:5) to afford **5** as a white solid (292 mg, 92%). ^1^H NMR (400 MHz, DMSO-d_*6*_) δ 7.90 (d, *J* = 7.4 Hz, 2H), 7.72 (d, *J* = 7.1 Hz, 2H), 7.42 (t, *J* = 7.1 Hz, 2H), 7.34 (t, *J* = 7.3 Hz, 2H), 7.26 (br, 1H), 6.99 (br, 1H), 4.29 (d, *J* = 6.3 Hz, 2H), 4.23 (t, *J* = 6.7 Hz, 1H), 3.55 (d, *J* = 5.8 Hz, 2H); ^13^C NMR (100 MHz, DMSO-d_*6*_,) δ 171.1, 156.5, 143.9, 140.7, 127.6, 127.1, 125.3, 120.1, 65.7, 46.7, 43.3; HRMS (ESI-TOF) calculated for C_17_H_17_N_2_O_3_ [M^+^H]^+^: 297.1234; found: 297.1249.

### Fmoc-Glycine Thioamide (6)

Lawesson reagent (404 mg, 1.0 mmol) was added to a solution of **5** (296 mg, 1.0 mmol) dissolved in dimethoxyethane (DME, 15 ml). The reaction was quenched by the addition of saturated sodium bicarbonate (NaHCO_3_) after stirring at room temperature for 12 h. The mixture was extracted successively with EtOAc, 5% (w/v) KHSO_4_, and brine. It was then dried over Na_2_SO_4_, filtered, passed through a short silica gel pad, and then concentrated under reduced pressure*. N*-Fmoc-glycine thioamide (**6**) was obtained as a white powder and used without further purification.

### Fmoc-Gly-Thz-OH (2)

Bromopyruvic acid (334 mg, 2.0 mmol) in DME (5 ml) was added dropwise at 0°C into a slurry of CaCO_3_ (600.5°mg, 3.0 mmol) and **6** (312.4 mg, 1.0 mmol) in DME (5 ml) with stirring. The mixture was allowed to gradually warm to room temperature over 12 h, which was then filtered and washed with DME. The solvent was removed *in vacuuo* and the remaining residue was dissolved in EtOAc, washed successively with 5% (w/v) KHSO_4_ and brine, and dried over Na_2_SO_4_. Recrystallization in EtOAc/hexane gave **7** as a white solid (277.8 mg, 73%). ^1^H NMR (400 MHz, DMSO-*d*_*6*_) δ 13.0 (br, 1H), 8.35 (s, 1H), 8.29 (t, J = 5.45 Hz, 1H), 7.89 (d, *J* = 7.3 Hz, 2H), 7.70 (d, *J* = 7.3 Hz, 2H), 7.42 (t, *J* = 7.2 Hz, 2H), 7.33 (t, *J* = 7.2 Hz, 2H), 4.48 (d, *J* = 5.8 Hz, 2H), 4.39 (d, *J* = 6.7 Hz, 2H), 4.25 (t, *J* = 6.4 Hz, 1H); ^13^C NMR (100 MHz, DMSO-*d*_*6*_) δ 170.8, 162.0, 156.4, 146.9, 143.7, 140.8, 128.5, 127.6, 127.1, 125.1, 120.1, 65.8, 46.7, 42.2; HRMS (ESI-TOF) calculated for C_20_H_16_N_2_O_4_SNa [M^+^Na]^+^: 403.0726; found: 403.0723.

### Fmoc-l-Valine Thioamide (8)

Compound **8** was synthesized using procedures similar to those described above for Fmoc-glycine thioamide (**6**). See Supplementary Material for details.

### Fmoc-l-Val-Thz-OEt (9)

Ethyl bromopyruvate (90% solution, 627 μl, 4.5 mmol) was added dropwise at 0°C to a solution of **8** (531 mg, 1.5 mmol) and 2,6-lutidine (524 μl, 4.5 mmol) in DME (5 ml) with stirring. The mixture was allowed to gradually warm to room temperature over 16 h. Trifluoroacetic anhydride (TFAA, 625 μl, 4.5 mmol) and 2,6-lutidine (524 μl, 4.5 mmol) in DME (2 ml) was added at 0°C and stirred at room temperature for 1 h. The reaction mixture was diluted with EtOAc, washed successively with saturated NaHCO_3_, 5% (w/v) KHSO_4_, and brine, dried over Na_2_SO_4_, and purified by silica gel column chromatography (*R*_*f*_ = 0.30, hexanes/EtOAc = 3:1) to yield **11** (648 mg, 96%) as an orange oil. ^1^H NMR (CDCl_3_, 400 MHz) δ 8.02 (s, 1H), 7.68 (d, *J* = 7.3 Hz, 2H), 7.54 (br, 2H), 7.32 (t, *J* = 7.3 Hz, 2H), 7.23 (t, *J* = 7.1 Hz, 2H), 5.86 (d, *J* = 9.0 Hz, 1H), 4.94 (t, *J* = 7.1 Hz, 1H), 4.40–4.33 (m, 4H), 4.16 (t, *J* = 5.5 Hz, 1H), 2.42–2.37 (m, 1H), 1.34 (t, *J* = 7.1 Hz, 3H), 0.93–0.88 (m, 6H); ^13^C NMR (CDCl_3_, 100 MHz) δ 172.0, 160.9, 155.8, 146.9, 143.4, 143.3, 140.9, 127.3, 126.7, 124.7, 119.6, 66.6, 61.0, 58.3, 46.8, 33.0, 19.1, 17.2, 14.0; HRMS (ESI-TOF) calculated for C_25_H_27_N_2_O_4_S [M^+^H]^+^: 451.1680, found: 451.1686.

### Me2-l-Val-Thz-OEt (10)

Compound **9** (450 mg, 1.0 mmol) was taken up in 20% (v/v) diethylamine in CH_2_Cl_2_ (10 ml), stirred at room temperature for 2 h, concentrated under reduced pressure, and then dissolved in methanol (5 ml). Formaldehyde solution (37% w/w) was added dropwise (245.7 μl, 3.0 mmol). Sodium cyanoborohydride (220 mg, 3.5 mmol) was added 40 min. later, and the resulting mixture was stirred at room temperature for another 15 h. The solvent was removed under reduced pressure and the residue was redissolved in EtOAc, which was then washed successively with sat. NaHCO_3_ and brine, dried over Na_2_SO_4_, and purified by silica gel column chromatography (*R*_*f*_ = 0.33, CH_2_Cl_2_/EtOAc = 9:1) to afford **10** (161 mg, 63%) as an orange oil. ^1^H NMR (CDCl_3_, 400 MHz) δ 8.09 (s, 1H), 4.35 (q, *J* = 7.1 Hz, 2H), 3.55 (d, *J* = 8.8 Hz, 1H), 2.17 (s, 1H), 2.14–2.09 (m, 1H), 1.33 (t, *J* = 7.1 Hz, 3H), 0.95 (d, *J* = 6.6 Hz, 3H), 0.76 (d, *J* = 6.6 Hz, 3H); ^13^C NMR (CDCl_3_, 100 MHz) δ 170.0, 161.2, 145.9, 127.1, 72.8, 61.1, 41.5, 29.9, 19.8, 18.6, 14.1; HRMS (ESI-TOF) calculated for C_12_H_21_N_2_O_2_S [M^+^H]^+^: 257.1329, found: 257.1318.

### Me2-l-Val-Thz-OH (3)

Compound **10** (231 mg, 0.9 mmol) was dissolved in tetrahydrofuran (2 ml) and cooled to 0°C. Lithium hydroxide (2 ml, 1M) was added, and the reaction mixture was stirred at room temperature for 1.5 h. It was neutralized at 0°C with 1N HCl and warmed to room temperature. The crude mixture was concentrated and purified by column chromatography using a manually packed column of C18 resins using a gradient of 0–5% (v/v) methanol. Compound **3** appeared as a colorless foam after solvent removal (213 mg, 92%). ^1^H NMR (CD_3_OD, 400 MHz) δ 8.51 (s, 1H), 4.91 (d, *J* = 7.9 Hz, 1H), 2.93 (s, 6H), 2.72–2.64 (m, 1H), 1.12 (d, *J* = 6.6 Hz, 3H), 0.91 (d, *J* = 6.5 Hz, 3H); ^13^C NMR (CD_3_OD, 100 MHz) δ 164.3, 162.2, 131.2, 71.6, 42.2, 30.3, 20.2, 18.2; HRMS (ESI-TOF) calculated for C_10_H_17_N_2_O_2_S [M^+^H]^+^: 229.1005, found: 229.1016. Compound **3** was then used as a typical amino acid building block in SPPS.

### Solid-Phase Peptide Synthesis

Standard Fmoc-based solid-phase peptide synthesis procedures were employed; see Supplementary Material for details.

### Macrocyclization

Upon completion of peptide synthesis, global deprotection and cleavage was performed by treating the resins with a trifluoroacetic acid (TFA) cocktail supplemented with 2.5% (v/v) of each triisopropylsilane and water. The resins were removed by filtration and TFA was dried under a gentle nitrogen stream. Cold hexane (approx. 50 ml) was added and the linear precursor of pagoamide A (**14**) crashed out as a light-yellow precipitate, which was collected by centrifugation at 4°C. PyAOP (208.5 mg, 0.4 mmol) and DIPEA (174.2 μl, 1.0 mmol) were added to **14** in DCM (25 ml) to effect macrocyclization. The crude product was concentrated under reduced pressure, dissolved in methanol, and purified by reversed-phase HPLC using a dual solvent system with a Hypersil GOLD aQ C18 column (250 (L) × 10 (ID) mm, 5 μm) at 8 ml/min, wherein solvent A and B are water and acetonitrile supplemented with 0.1% (v/v) of formic acid, respectively. The desired macrocyclic product **1** eluted at 40%B. HRMS (ESI-TOF) calculated for C_51_H_68_N_11_O_12_S_2_ [M^+^H]^+^: 1,090.449, found: 1,090.451. See Supplementary Material for analytical data, including HPLC traces, MALDI-MS (reaction monitoring), and NMR and HRMS spectra.

### Antimicrobial Assays

Microbes were grown from single colonies on agar plates into LB (bacteria) and YPD broths (*Candida albicans*). Assays were carried out in 96-well microtiter plates to determine the minimum inhibitory concentrations (MICs) of **1** against various microbial pathogens by using the broth microdilution method. Briefly, DMSO stock solution of synthetic **1** (12.8 mg/ml) was added to the first well of each row and 1/2× serially diluted across the plate. Wells 11 and 12 were free of **1** (positive control) and free of microbe (negative control), respectively. Overnight microbial cultures were diluted 5,000-fold and used as the inocula, such that the final volume in all wells were 100 μl with the concentrations of **1** ranging from 64 to 0.125 μg/ml in each row. The plate was incubated statically at 30°C for 24 h. The lowest concentration of **1** that led to inhibition of microbial growth was recorded as the MIC.

## Results

Pagoamide A (**1**) is a cyclic depsipeptide made of 11 amino acids, three of which are d-amino acids. While SPPS is an obvious choice for the construction of its backbone, as both l- and d-Fmoc building blocks are readily available from commercial venders, a number of features in **1** call for expanding the scope of synthesis beyond simple amino acid coupling in conventional SPPS. These features include two thiazole rings, the dimethylated *N*-terminus, and the macrocyclic structure.

**SCHEME 1:**
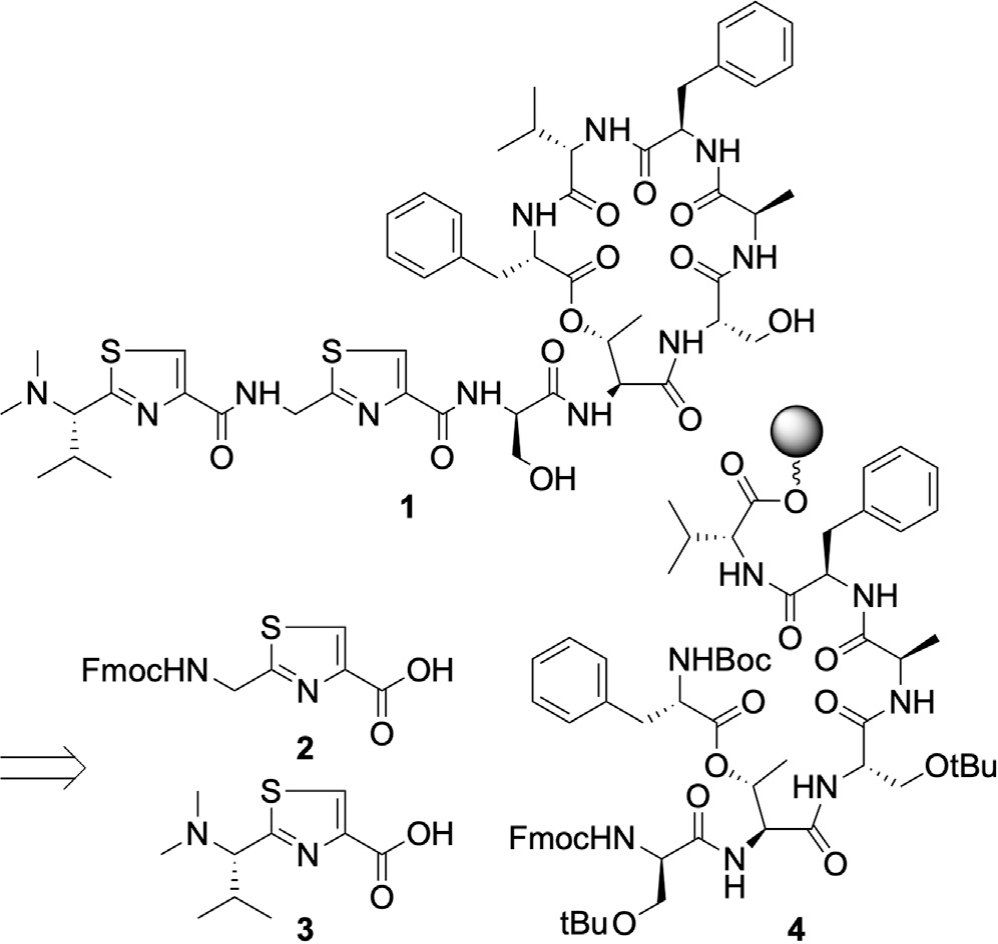
Retrosynthetic analysis of pagoamide A.

In our retrosynthetic plan (Scheme 1), each thiazole moiety will be incorporated as a pre-made building block that spans the length of two amino acids. In this strategy, because the dimethylated *N*-terminus of **1** is part of a thiazole building block, *N*,*N*-dimethylation will be performed after thiazole formation and before the use of this building block in SPPS. Total syntheses of cyclic depsipeptides reported in the literature mostly opt to construct ester bonds early in the synthetic scheme rather than using its formation as the final macrocyclization step (Lam et al., 2013; Lohani et al., 2015). This is because amines, as opposed to the less nucleophilic hydroxyl groups, are generally a superior choice as the site to conclude macrocycle formation. We therefore planned to build **4** as a key intermediate.

**SCHEME 2:**
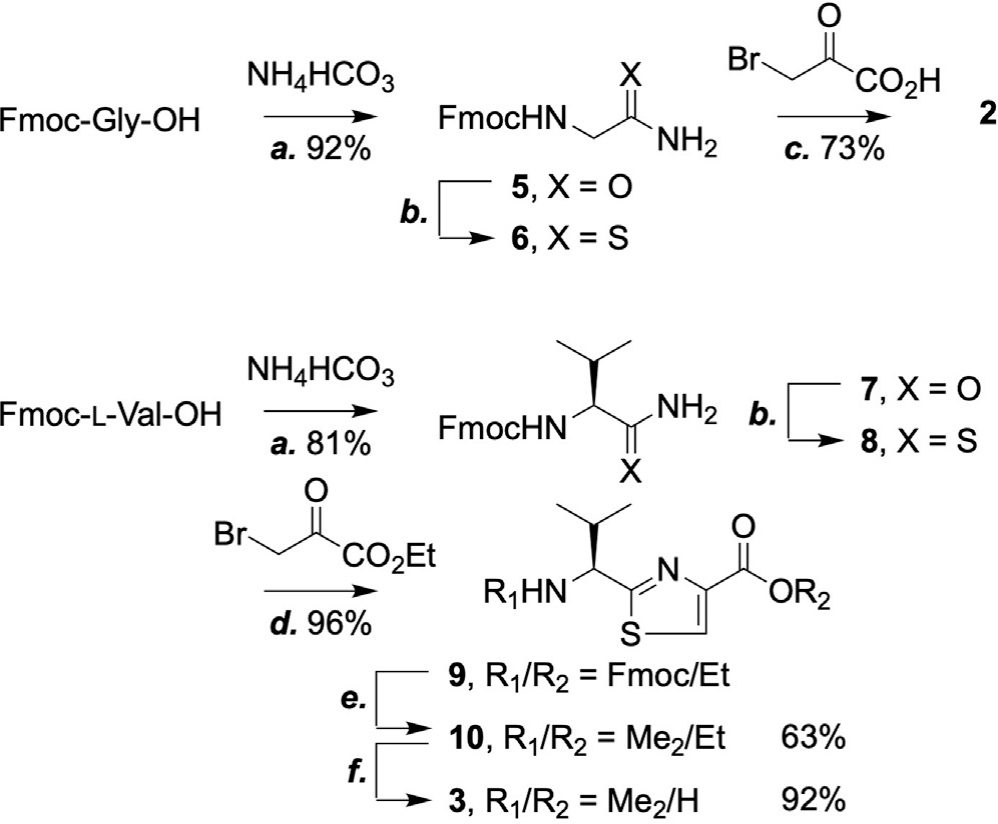
Synthesis of thiazole building blocks. Reagents and conditions: **(a)** Ammonium bicarbonate (1 equiv.), HBTU (1.5 equiv.), DIPEA (2 equiv.), THF, 25°C; **(b)** Lawesson reagent (1 equiv.), DME, 25°C; **(c)** Bromopyruvic acid (1 equiv.), CaCO_3_ (3 equiv.), DME, 25°C; **(d)** Ethyl bromopyruvate (3 equiv.), 2,6-lutidine (3 equiv.), DME, 0–25°C then TFAA (3 equiv.), 2,6-lutidine (3 equiv.), 0–25°C; **(e)** Formaldehyde solution (37% w/w, 3 equiv.), sodium cyanoborohydride (3.5 equiv.), 25°C; **(f)** LiOH (2 ml, 1 M), THF, 25°C.

We started by constructing the thiazole building blocks **2** and **3** (Scheme 2). *N*-Fmoc-protected glycine (Fmoc-Gly-OH) was first converted to the amide (**5**) and then, via the use of Lawesson reagent, (Thomsen et al., 1984) the thioamide (**6**). Hantzsch thiazole synthesis afforded **2** with satisfactory yield (Singh et al., 2012; Fenner et al., 2016; Aihara et al., 2017). The same workflow turned Fmoc-l-valine into the corresponding thiazole (**9**), except for the use of trifluoroacetic anhydride (TFAA) on the condensation product of ethyl bromopyruvate and **8** to promote dehydration. Once the Fmoc protecting group was removed from **9**, two methyl groups were installed onto the *N*-terminus by reductive amination with an excess amount of sodium cyanoborohydride and formaldehyde to give **10**, which was hydrolyzed to afford **3**. Both compounds **2** and **3** are now ready-to-use SPPS building blocks.

**SCHEME 3:**
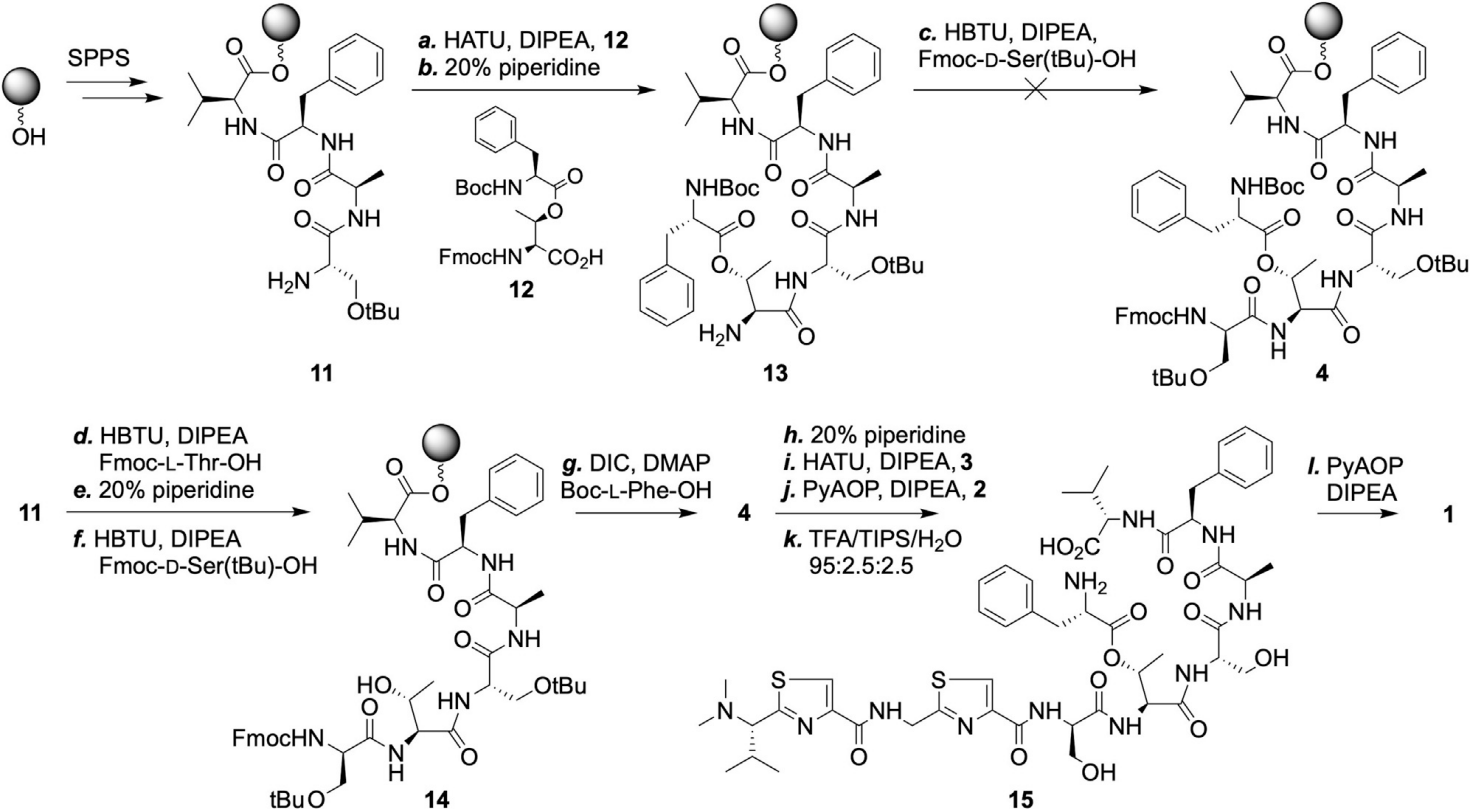
SPPS based synthesis of pagoamide A (1). Reagents and conditions: Standard SPPS procedures were carried out throughout the synthesis of pagoamide A (see Supplementary Material). Amide couplings were carried out in steps ***a***, ***c***, ***d***, ***f***, ***i***, and ***j***, except for the use of varying coupling reagents; Fmoc groups were removed in steps ***b***, ***e***, ***h***, and after step ***j***. ***g.*** DIC (10 equiv.), DMAP (2 equiv.), 25°C; ***k.*** TFA cocktail, 25°C; ***l.*** PyAOP (4 equiv.), DIPEA (10 equiv.), DCM, 25°C.

A critical part of our plan was to rely on the formation of an ester bond as the final macrocyclization step. With this in mind, we attempted the synthesis of intermediate **4** en route a pre-made threonine-phenylalanine dimer (**12**) (Scheme 3). The coupling of **12** to extend the peptide chain on solid-support proceeded without difficulty. The very next coupling, however, was sluggish and saw no improvement in yield after repeated couplings or the use of alternative reagents. This prompted us to explore a different sequence in constructing **4**, wherein a threonine building block without side-chain protection was incorporated as the sixth residue, followed by the coupling of a standard serine building block. Next, *N*-Boc-protected phenylalanine was activated by *N*,*N*’-diisopropylcarbodiimide and installed successfully onto the exposed threonine side-chain hydroxyl group. Subsequent couplings of **3** and **2** proceeded cleanly.

Trifluoroacetic acid cocktail treatment resulted in global deprotection and peptide cleavage off of the resins. The linear precursor of pagoamide A (**14**) was collected after cold hexane precipitation and subjected to macrocyclization without further purification. This reaction was monitored by MALDI-mass spectrometry and, to our delight, no signal of **14** was observed when four equivalents of the phosphonium coupling reagent PyAOP was used (see Supplementary Material). The amide bond formation reaction between the amine of the Phe_11_ and the carboxylate of the Val_10_ residues was essentially complete after 24 h. The HPLC purified final product was confirmed by high resolution ESI-MS. ^1^H NMR spectrum of synthetic **1** matches that obtained from the culture extracts of *Derbesia* sp., thereby confirming the reported stereochemistry assignment (see Supplementary Material for analytical characterizations) (Li et al., 2020).

The isolation of **1** from crude extracts was guided by a MS-based molecular network and a calcium oscillation assay (Yang et al., 2013; Wang et al., 2016). However, once purified, **1** turned out to be inactive in this very assay and showed no cytotoxicity against the H-460 human lung cancer cell line. No other bioactivity was reported. We were able to obtain sufficient amount of **1** by total synthesis for antimicrobial assays and performed a systematic evaluation of its antimicrobial potential (M07-A9, 2012). Our assay included the ESKAPE pathogens (Mulani et al., 2019) – *Enterococcus faecalis*, *Staphylococcus aureus*, *Klebsiella pneumoniae*, *Acinetobacter baumannii*, *Pseudomonas baumannii*, and *Enterobacter* spp. – a panel of highly virulent bacteria whose antibiotic resistant strains are the major cause of fatal infections in humans around the world. Also included in our assay are *Bacillus subtilis* and *Escherichia coli*, model organisms for Gram-positive and Gram-negative bacteria, respectively, as well as a common fungal pathogen *Candida albicans*. In this initial round of screening, our results showed that **1** has only moderate activity (64 μg/ml) against *Bacillus subtilis* (Table 1).

**TABLE 1:** Pagoamide A (1) was tested against a number of common pathogens.

Name of pathogen	MIC^a^ (μg/ml)
*Bacillus subtilis* BCRC 10614	64
*Escherichia coli* DH5α	>
*Enterococcus faecalis* BCRC 10789	>
*Staphylococcus aureus* BCRC 11863	>
*Klebsiella pneumoniae* BCRC 11546	>
*Acinetobacter baumannii* BCRC 10591	>
*Pseudomonas aeruginosa* BCRC 11864	>
*Enterobacter cloacae* BCRC 10401	>
*Candida albicans* BCRC 21538	>

a The highest concentration tested was 64 μg/ml.

## Discussion

We reasoned that one could combine the convenience of peptide synthesis (performed on solid support) and the versatility of conventional organic synthesis (performed in solution) into a powerful new synthetic platform. This platform would be particularly useful in the synthesis of natural products of peptide backbones. We put this concept into practice and report the synthesis of **1**, a natural product with complex structural features on top of a peptide backbone, whose thiazole units, one of which was dimethylated, were constructed in solution into ready-to-use building blocks. The entire linear precursor was assembled on-resin and then macrocyclized off-resin to afford **1**. The approach presented herein is modular and broadly applicable to the synthesis of peptide natural products that contain heterocycles, *N*-methylations, and backbone macrocyclization, all of which are commonly seen structural features in peptide natural products.

A separate total synthesis of pagoamide A was reported by the Ye group after we submitted this manuscript (Wu et al., 2021).

## Data Availability

The original contributions presented in the study are included in the article/Supplementary Material, further inquiries can be directed to the corresponding author.

## References

[B1] Aihara K. Inokuma T. Jichu T. Lin Z. Fu F. Yamaoka K. (2017). Cysteine-free intramolecular ligation of *N*-sulfanylethylanilide peptide using 4-mercaptobenzylphosphonic acid: synthesis of cyclic peptide trichamide. Synlett 28, 1944–1949.

[B2] Arnison P. G. Bibb M. J. Bierbaum G. Bowers A. A. Bugni T. S. Bulaj G. (2013). Ribosomally Synthesized and post-translationally Modified Peptide Natural Products: Overview and Recommendations for a Universal Nomenclature. Nat. Prod. Rep. 30, 108–160. 10.1039/c2np20085f 23165928 PMC3954855

[B3] Coin I. Beyermann M. Bienert M. (2007). Solid-phase Peptide Synthesis: from Standard Procedures to the Synthesis of Difficult Sequences. Nat. Protoc. 2, 3247–3256. 10.1038/nprot.2007.454 18079725

[B4] Fenner S. Wilson Z. E. Ley S. V. (2016). The Total Synthesis of the Bioactive Natural Product Plantazolicin A and its Biosynthetic Precursor Plantazolicin B. Chem. Eur. J. 22, 15902–15912. 10.1002/chem.201603157 27619732

[B5] Fischbach M. A. Walsh C. T. (2006). Assembly-Line Enzymology for Polyketide and Nonribosomal Peptide Antibiotics: Logic, Machinery, and Mechanisms. Chem. Rev. 106, 3468–3496. 10.1021/cr0503097 16895337

[B6] Lam H. Y. Zhang Y. Liu H. Xu J. Wong C. T. T. Xu C. (2013). Total Synthesis of Daptomycin by Cyclization via a Chemoselective Serine Ligation. J. Am. Chem. Soc. 135, 6272–6279. 10.1021/ja4012468 23560543

[B7] Li Y. Yu H.-B. Zhang Y. Leao T. Glukhov E. Pierce M. L. (2020). Pagoamide A, a Cyclic Depsipeptide Isolated from a Cultured marine Chlorophyte, *Derbesia* sp., Using MS/MS-based Molecular Networking. J. Nat. Prod. 83, 617–625. 10.1021/acs.jnatprod.9b01019 31916778 PMC7210564

[B8] Lohani C. R. Taylor R. Palmer M. Taylor S. D. (2015). Solid-phase Total Synthesis of Daptomycin and Analogs. Org. Lett. 17, 748. 10.1021/acs.orglett.5b00043 25634084

[B9] M07-A9 (2012). Methods for Dilution Antimicrobial Susceptibility Tests for Bacteria that Grow Aerobically; Approved Standard - Ninth Edition. Wayne, PA: Clinical and Laboratory Standards Institute.

[B10] McIntosh J. A. Donia M. S. Schmidt E. W. (2009). Ribosomal Peptide Natural Products: Bridging the Ribosomal and Nonribosomal Worlds. Nat. Prod. Rep. 26, 537–559. 10.1039/b714132g 19642421 PMC2975598

[B11] Mulani M. S. Kamble E. E. Kumkar S. N. Tawre M. S. Pardesi K. R. (2019). Emerging Strategies to Combat ESKAPE Pathogens in the Era of Antimicrobial Resistance: A Review. Front. Microbiol. 10, 539. 10.3389/fmicb.2019.00539 30988669 PMC6452778

[B12] Newman D. J. Cragg G. M. (2020). Natural Products as Sources of New Drugs over the Nearly Four Decades from 01/1981 to 09/2019. J. Nat. Prod. 83, 770–803. 10.1021/acs.jnatprod.9b01285 32162523

[B13] Singh E. K. Ramsey D. M. McAlpine S. R. (2012). Total Synthesis of Trans,trans-Sanguinamide B and Conformational Isomers. Org. Lett. 14, 1198–1201. 10.1021/ol203290n 22356651

[B14] Takayama R. Hayakawa S. Hinou H. Albericio F. Garcia-Martin F. (2018). Further Applications of Classical Amide Coupling Reagents: Microwave-Assisted Esterification on Solid Phase. J. Pep Sci. 24, e3111. 10.1002/psc.3111 30009478

[B15] Thomsen I. Clausen K. Scheibye S. Lawesson S. O. (1984). Thiation with 2,4-Bis(4-Methoxyphenyl)-1,3,2,4- Dithiadiphosphetane 2,4-Disulfide: N-Methylthiopyrrolidone. Org. Synth. 62, 158. 10.15227/orgsyn.062.0158

[B16] Wang M. Carver J. J. Phelan V. V. Sanchez L. M. Garg N. Peng Y. (2016). Sharing and Community Curation of Mass Spectrometry Data with Global Natural Products Social Molecular Networking. Nat. Biotechnol. 34, 828–837. 10.1038/nbt.3597 27504778 PMC5321674

[B17] Wu F. Yu J. Meng J. Guo Y. Ye T. (2021). Total Synthesis of Pagoamide A. Molecules 26, 4224. 10.3390/molecules26144224 34299497 PMC8307129

[B18] Yang J. Y. Sanchez L. M. Rath C. M. Liu X. Boudreau P. D. Bruns N. (2013). Molecular Networking as a Dereplication Strategy. J. Nat. Prod. 76, 1686–1699. 10.1021/np400413s 24025162 PMC3936340

