# Supplementary material for: Total Synthesis and Antimicrobial Evaluation of Pagoamide A
Source: Front Chem. 2021 Sep 14;9:741290. doi: 10.3389/fchem.2021.741290 (PMC8476950; doi:10.3389/fchem.2021.741290)
Supplement: Supplementary file 1 [file DataSheet2.pdf]

Scheme 1. Retrosynthetic analysis of pagoamide A.

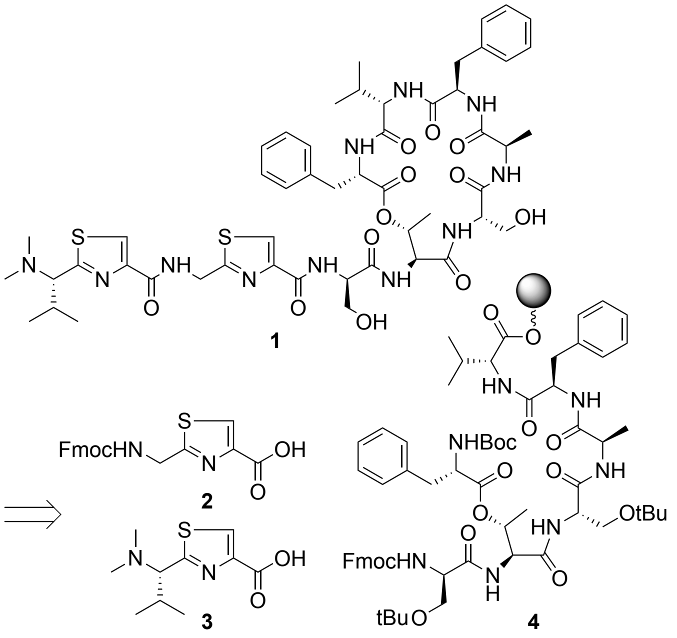

Scheme 2. Synthesis of thiazole building blocks.

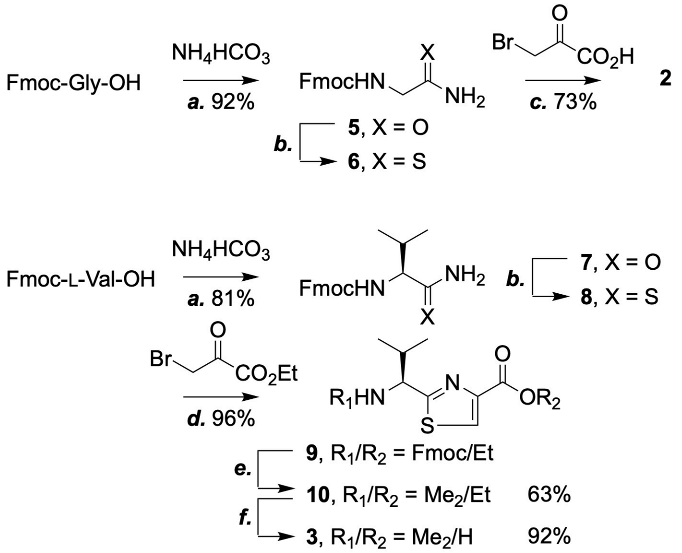

Reagents and conditions: ***a.*** Ammonium bicarbonate (1 equiv.), HBTU (1.5 equiv.), DIPEA (2 equiv.), THF, 25 ºC; ***b.*** Lawesson reagent (1 equiv.), DME, 25 ºC; ***c.*** Bromopyruvic acid (1 equiv.), $CaCO_3$ (3 equiv.), DME, 25 ºC; ***d.*** Ethyl bromopyruvate (3 equiv.), 2,6-lutidine (3 equiv.), DME, 0 to 25 ºC then TFAA (3 equiv.), 2,6-lutidine (3 equiv.), 0 to 25 ºC; ***e.*** Formaldehyde solution (37% w/v, 3 equiv.), sodium cyanoborohydride (3.5 equiv.), 25 ºC; ***f.*** LiOH (2 mL, 1 M), THF, 25 ºC.

Scheme 3. SPPS based synthesis of pagoamide A (1)

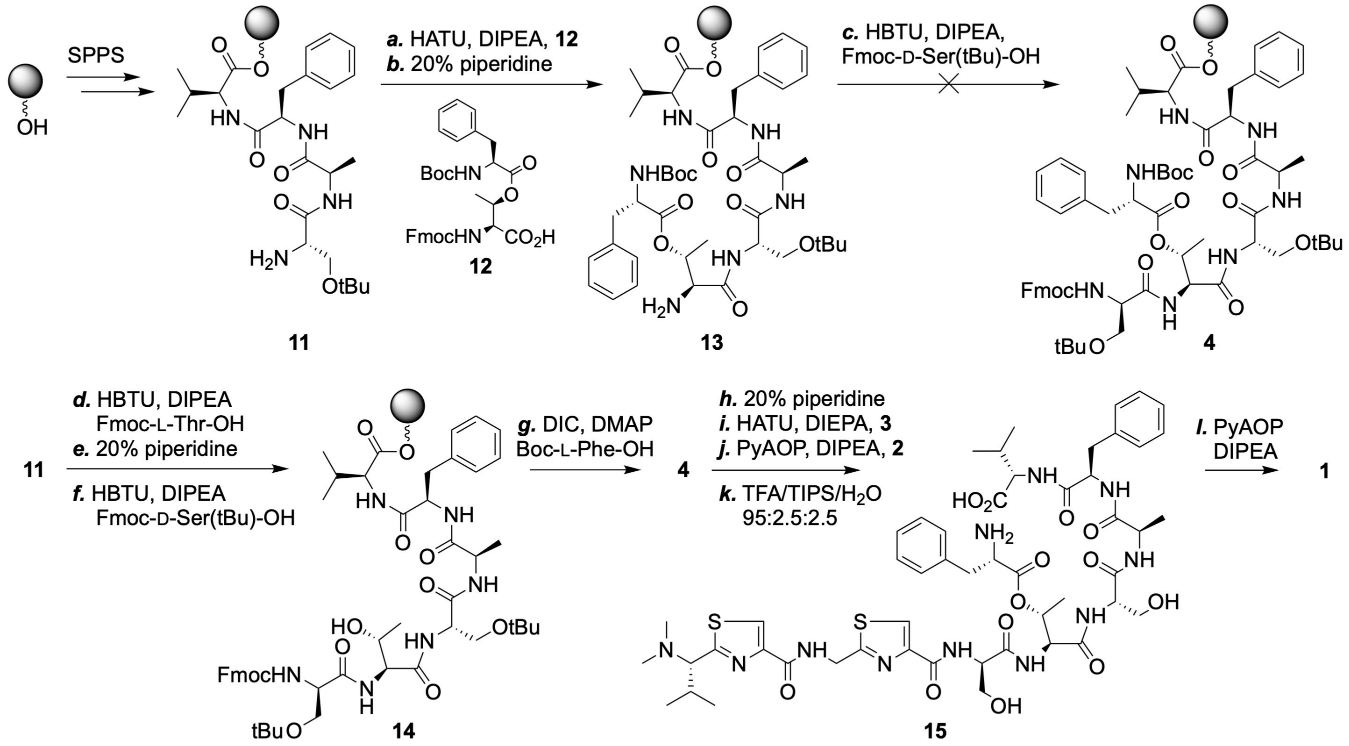

Reagents and conditions: Standard SPPS procedures were carried out throughout the synthesis of pagoamide A (see Supplementary Materials). Amide couplings were carried out in steps ***a***, ***c***, ***d***, ***f***, ***i***, and ***j***, except for the use of varying coupling reagents; Fmoc groups were removed in steps ***b***, ***e***, ***h***, and after step ***j***. ***g.*** DIC (10 equiv.), DMAP (2 equiv.), 25 °C; ***k.*** TFA cocktail, 25 °C; ***l.*** PyAOP (4 equiv.), DIPEA (10 equiv.), DCM, 25 °C.

Table 1. Pagoamide A (1) was tested against a number of common pathogens.

Name of pathogen	MIC[a] (μg/mL)
Bacillus subtilis BCRC 10614	64
Escherichia coli DH5α	>
Enterococcus faecalis BCRC 10789	>
Staphylococcus aureus BCRC 11863	>
Klebsiella pneumoniae BCRC 11546	>
Acinetobacter baumannii BCRC 10591	>
Pseudomonas aeruginosa BCRC 11864	>
Enterobacter cloacae BCRC 10401	>
Candida albicans BCRC 21538	>

[a] The highest concentration tested was 64 μg/mL

This is a provisional file, not the final typeset article
