# Supplementary material for: Total Synthesis and Antimicrobial Evaluation of Pagoamide A
Source: Front Chem. 2021 Sep 14;9:741290. doi: 10.3389/fchem.2021.741290 (PMC8476950; doi:10.3389/fchem.2021.741290)

Supplementary Material

TABLE of CONTENTS

General Procedures for SPPS … 2

- Resin loading
- Amino acid coupling
- Global deprotection and cleavage

Synthesis of Noncanonical Building Blocks … 3

- Fmoc-L-Val-NH_2 (**7**)
- Fmoc-L-valine thioamide (**8**)
- Fmoc-L-Thr-OBn (**16**)
- Fmoc-L-Thr(*N*-Boc-Phe)-OBn (**17**)
- Fmoc-L-Thr(Boc-Phe)-OH (**12**)

Supplementary Figures – HPLC and MS … 6

- Macrocyclization reaction monitored by MALDI-MS
- HPLC trace of pure synthetic pagoamide A (**1**)
- High resolution mass spectrum of synthetic pagoamide A (**1**)

Supplementary Figures – NMR Spectra … 8

- ^{1}H NMR spectra: pagoamide A isolated from *Derbesia* sp. vs. synthetic compound
- ^{1}H and ^{13}C NMR spectra of Fmoc-Gly-NH_2 (**5**)
- ^{1}H and ^{13}C NMR spectra of Fmoc-Gly-Thz-OH (**2**)
- ^{1}H and ^{13}C NMR spectra of Fmoc-L-Val-NH_2 (**7**)
- ^{1}H and ^{13}C NMR spectra of Fmoc-L-valine thioamide (**8**)
- ^{1}H and ^{13}C NMR spectra of Fmoc-L-Val-Thz-OEt (**9**)
- ^{1}H and ^{13}C NMR spectra of Me_2-L-Val-Thz-OEt (**10**)
- ^{1}H and ^{13}C NMR spectra of Me_2-L-Val-Thz-OH (**3**)
- ^{1}H and ^{13}C NMR spectra of Fmoc-L-Thr-OBn (**16**)
- ^{1}H and ^{13}C NMR spectra of Fmoc-L-Thr(*N*-Boc-Phe)-OBn (**17**)
- ^{1}H and ^{13}C NMR spectra of Fmoc-L-Thr(*N*-Boc-Phe)-OH (**12**)

GENERAL PROCEDURES for SPPS

Resin loading. The linear precursor of pagoamide A was synthesized on Wang resins (0.1 mmol). Prior to starting SPPS, Wang resins were swollen in DMF for 0.5 h. The first amino acid building block (Fmoc-L-Val-OH, 170 mg, 0.5 mmol) was activated with *N*,*N'*-diisopropyl-carbodiimide (DIC, 63 mg, 0.5 mmol) in DMF (4 mL). 4-Dimethylaminopyridine (DMAP, 6.1 mg, 0.05 mmol) was added to the DMF solution and poured over the resins. The slurry was shaken at room temperature for 2 h. After draining the loading mixture, the resins were washed thoroughly with DMF (approx. 4 mL, 5×). The loading step was repeated once. The loading yield was quantitated spectroscopically based on the amount of Fmoc adduct as a result of microcleavage by 20% (v/v) piperidine in DMF, wherein ε = 6,000 M^{-1} cm^{-1} at 301 nm in DMF.

A typical round of **amino acid coupling** entails the following two steps.

Fmoc removal. The Fmoc protecting group was removed by treating the resins with 20% (v/v) piperidine in DMF for 8 min (3×). The resin was then washed with DMF (approx. 4 mL, 5×).

Coupling. *N*-Fmoc protected amino acid (approx. 0.3 mmol, *i.e.*, 3 equivalents relative to the amount of resins used), HBTU (3 eq.), HOBt (3 eq.), and DIPEA (10 eq.) were mixed in DMF (3 mL), add to the resins, and then shaken at 120 rpm at room temperature for 45 min. After draining the solution, the resins were washed with DMF (approx. 4 mL, 5×).

Special note. The Fmoc-Gly-Thz-OH building block (**2**) was activated by using the same procedure as described above, except that HATU, which reacts faster with less epimerization, was used as the activating reagent. Tertiary amines are known to react with uronium reagents, albeit slowly, and therefore we avoided the use of HATU and HBTU in the activation of the Me_2-Val-Thz-OH building block (**3**). PyAOP was used as the activation reagent instead.

Global deprotection and cleavage. Upon completion of SPPS, the resins were washed several times with DMF and DCM. Global deprotection and cleavage was performed using a TFA cocktail containing 2.5% of each triisopropylsilane and water at room temperature for 2 h. The resins were removed by filtration and the TFA cocktail was dried under a gentle nitrogen stream. Cold hexane (50 mL) was added and the crude peptide crashed out as a light-yellow precipitate, which was collected by centrifugation at 4 °C.

SYNTHESIS of NONCANONICAL BUILDING BLOCKS

Fmoc-L-Val-NH$_2$ (7)

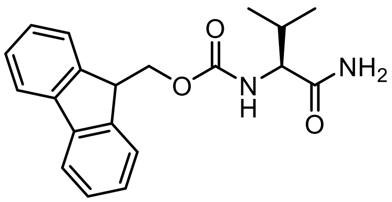

Fmoc-Val-OH (1.697 g, 5.0 mmol), pyridine (242 μL, 3.0 mmol), and ammonium bicarbonate (500 mg, 6.3 mmol) were dissolved in DMF (7 mL). Boc$_2$O (1.49 mL, 6.5 mmol) was added to this mixture and stirred at room temperature for 16 h. Fmoc-Val-NH$_2$ (**7**) was obtained as a white precipitate upon the addition of 1 N HCl. The precipitate was washed and **7** was obtained as a white solid (1.590 g, 94%). ^{1}H NMR (DMSO-*d6*, 400 MHz) δ 7.89 (d, J = 7.5 Hz, 2H), 7.75 (m, 2H), 7.42 (t, J = 7.3 Hz, 2H), 7.25 – 7.30 (m, 4H), 7.03 (br, 1H), 4.30 – 4.23 (m, 3H), 3.84 (t, J = 7.9 Hz , 1H), 1.97 (m, 1H), 0.90 – 0.86 (m, 6H); ^{13}C NMR (DMSO-*d6*, 100 MHz) δ 173.2, 156.1, 143.9, 143.8, 140.7, 127.6, 127.0, 125.3, 120.0, 65.6, 60.1, 46.7, 30.1, 19.3, 18.1; HRMS (ESI-TOF) calculated for $C_{20}H_{22}N_2O_2Na$ $[M+Na]^+$: 361.1523, found: 361.1530.

Fmoc-L-valine thioamide (8)

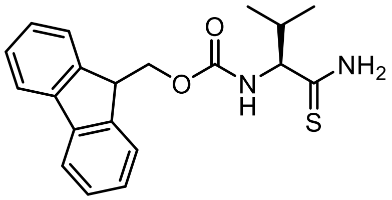

Lawesson reagent (809 mg, 2.0 mmol) was added to a solution of **7** (338 mg, 1.0 mmol) in dimethoxyethane, and the reaction was allowed to proceed at 40 °C for 18 h before it was quenched by saturated sodium bicarbonate. The mixture was extracted using EtOAc, washed successively with 5% (w/v) potassium bisulfate and brine, dried over sodium sulfate, and purified by silica gel column chromatography (R_f = 0.38, 2.5% (v/v) MeOH in CH_2Cl_2) to give **8** as a white foam (322 mg, 91%). ^{1}H NMR (400 MHz, $CDCl_3$) δ 8.45 (br, 1H), 8.29 (br, 1H), 7.76 (d, J = 7.4 Hz, 2H), 7.60 (t, J = 8.0 Hz, 2H), 7.40 (m, 2H), 7.32 (m, 2H), 6.01 (d, J = 8.8 Hz, 1H), 4.37 (m, 2H), 4.21 (t, J = 6.9 Hz, 1H), 2.14 (m, 1H), 1.01 (d, J = 6.0 Hz, 3H), 0.97 (d, J = 6.5 Hz, 3H); ^{13}C NMR (100 MHz, $CDCl_3$) δ 208.5, 156.4, 143.4, 143.3, 141.0, 127.5, 126.9, 124.8, 119.8, 67.0, 65.2, 46.8, 33.0, 19.2, 18.1; HRMS (ESI-TOF) calculated for $C_{20}H_{22}N_2O_2SNa$ $[M+Na]^+$: 377.1276; found: 377.1294.

Fmoc-L-Thr-OBn (16)

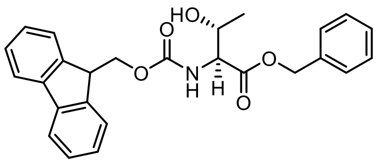

Benzylbromide (238 μL, 2.0 mmol) was added to a solution of Fmoc-Thr-OH (360 mg, 1.0 mmol) and Cs_2CO_3 (667 mg, 2.05 mmol) in DMF (5 mL) at room temperature, and the mixture was stirred for 3 h. The reaction was quenched by adding saturated NH_4Cl and extracted with EtOAc. The EtOAc extract was dried over Na_2SO_4, concentrated under reduced pressure, and purified using silica gel column chromatography (R_f = 0.2, hexane/EtOAc = 2:1) to give **16** as a white solid (345 mg, 80%). ^{1}H NMR (400 MHz, $CDCl_3$) δ 7.80 (d, J = 7.5 Hz, 2H), 7.65 (d, J = 7.2 Hz, 2H), 7.43 (t, J = 7.4 Hz, 2H), 7.37 – 7.31 (m, 5H), 5.93 (d, J = 8.9 Hz, 1H), 5.24 (s, 2H), 4.46 – 4.43 (m, 4H), 4.26 (t, J = 6.7 Hz, 1H), 2.69 (br, 1H), 1.28 (d, J = 6.5 Hz, 3H); ^{13}C NMR (100 MHz, $CDCl_3$) δ 171.1, 156.8, 143.8, 143.6, 141.2, 135.2, 128.6, 128.4, 128.1, 127.7, 127.0, 125.1, 67.9, 67.3, 67.2, 59.3, 47.1, 19.9; HRMS (ESI-TOF) calculated for $C_{26}H_{25}NO_5Na$ $[M+Na]^+$: 454.1625; found: m/z 454.1622.

Fmoc-L-Thr(*N*-Boc-Phe)-OBn (17)

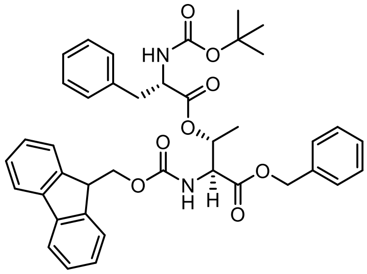

EDC (443 μL, 2.5 mmol) and DMAP (61 mg, 0.5 mmol) were added to a solution of Boc-Phe-OH (318 mg, 1.1 mmol) and **16** (432 mg, 1.0 mmol) in CH_2Cl_2 at room temperature. The solution was stirred overnight and then washed successively with 1 M HCl, 1 M NaOH, and brine. The organic extract was dried over Na_2SO_4, concentrated under reduced pressure, purified by silica gel column chromatography (R_f = 0.35, hexane/EtOAc = 3:1) to afford **17** as a white foam (212.1 mg, 56%). ^{1}H NMR (400 MHz, $CDCl_3$) δ 7.83 (d, J = 7.5 Hz, 2H), 7.71 (d, J = 7.3 Hz, 2H), 7.47 – 7.39 (m, 10H), 7.31 (t, J = 7.2 Hz, 2H), 7.22 (t, J = 8.9 Hz, 2H), 5.63 (d, J = 9.2 Hz, 1H), 5.50 (d, J = 4.6 Hz, 1H), 5.26 – 5.17 (m, 3H), 4.63 (d, J = 9.3 Hz, 1H), 4.54 – 4.48 (m, 3H), 4.30 (t, J = 6.6 Hz, 1H), 3.05 (d, J = 7.6 Hz, 2H), 1.52 (s, 9H), 1.25 (d, J = 6.1 Hz, 3H); ^{13}C NMR (100 MHz, $CDCl_3$) δ 170.9, 169.3,

156.6, 155.0, 143.9, 143.7, 141.3, 136.1, 135.1, 129.2, 128.6, 128.5, 127.7, 127.1, 125.1, 120.0, 79.9, 71.7, 67.7, 67.2, 57.5, 54.5, 47.2, 38.1, 28.3, 16.7; HRMS (ESI-TOF) calculated for $C_{40}H_{42}N_2O_8$ Na $[M+Na]^+$: 701.2833; found 701.2839.

Fmoc-L-Thr(Boc-Phe)-OH (12)

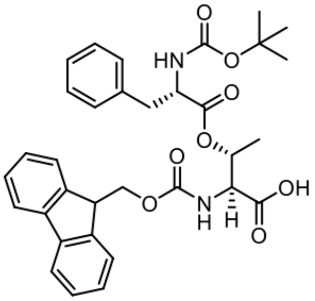

Palladium on charcoal (Pd/C, 10% (w/v) 28 mg) was added to a solution of **17** (272 mg, 0.4 mmol) in MeOH at room temperature. The flask was flushed with hydrogen for three times and stirred at room temperature overnight under a hydrogen-filled baloon. The slurry was filtered through a pad of Celite and washed with MeOH, concentrated under reduced pressure, and purified by silica gel column chromatography (R_f = 0.33, CH_2Cl_2/MeOH = 9:1) to give **12** as white solid (122.4 mg, 52%). ^{1}H NMR (400 MHz, CD_3OD) δ 7.69 (d, J = 7.4 Hz, 2H), 7.59 (d, J = 7.0 Hz, 2H), 7.29 (t, J = 7.4 Hz, 2H), 7.23 – 7.11 (m, 7H), 5.36 (s, 1H), 4.40 (t, J = 8.4 Hz, 1H), 4.30 (t, J = 6.1 Hz, 2H), 4.19 (s, 1H), 4.13 (t, J = 6.3 Hz, 1H), 3.04 – 2.99 (m, 1H), 2.82 (t, J = 10.9 Hz, 1H), 1.30 (s, 9H), 1.13 (d, J = 5.3 Hz,3H); ^{13}C NMR (100 MHz, CD_3OD) δ 171.3, 157.4, 156.3, 143.8, 143.6, 141.1, 136.8, 128.9, 127.9, 127.3, 126.7, 126.3, 124.7, 124.6, 119.5, 79.2, 72.3, 66.5, 59.4, 55.2, 37.5, 27.2, 16.1; HRMS (ESI-TOF) calculated for $C_{33}H_{36}N_2O_8Na$ $[M+Na]^+$: 611.2360; found: 611.2364.

SUPPLEMENTARY FIGURES – HPLC and MS

Monitor macrocyclization reaction by MALDI-MS

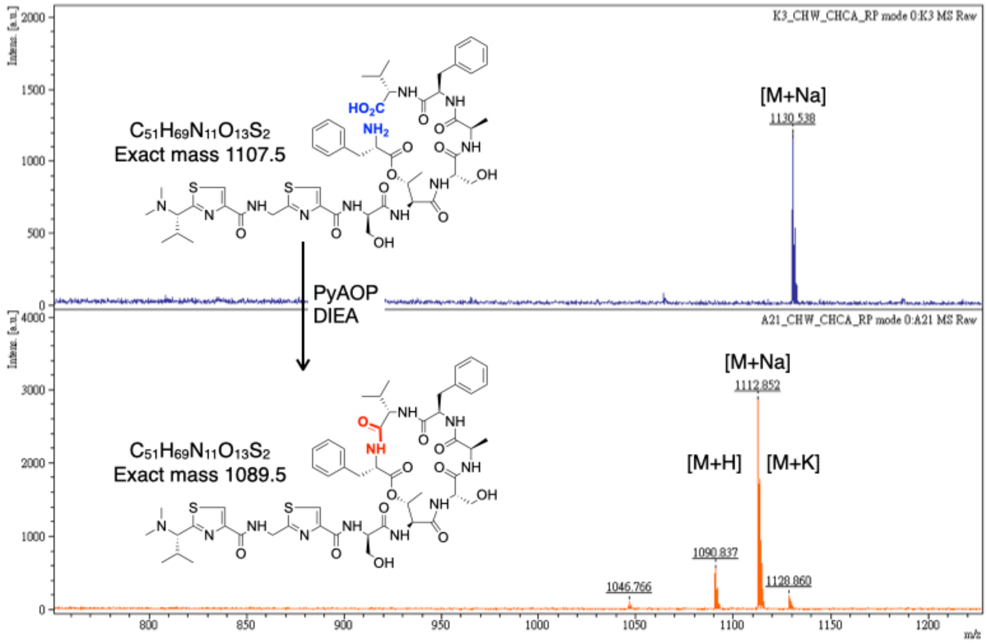

HPLC trace of pure synthetic pagoamide A (**1**)

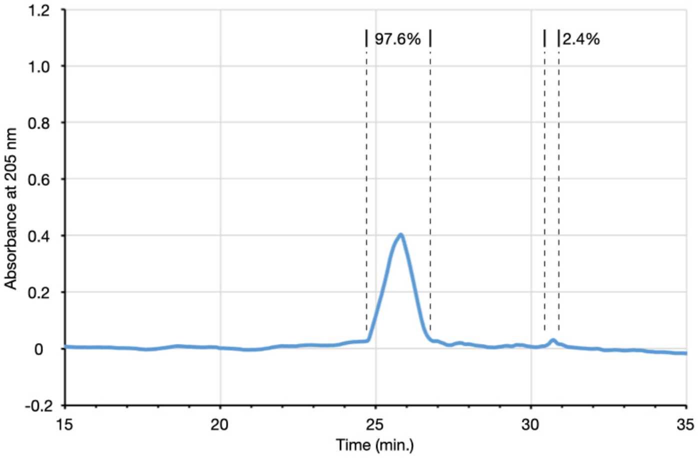

High resolution mass spectrum of synthetic pagoamide A (**1**)

Meas. m/z	#	Ion Formula	m/z	err [mDa]	err [ppm]	mSigma	Score	rdb	e⁻ Conf	N-Rule
1090.4507	1	C51H68N11O12S2	1090.4485	2.2	2.0	12.6	100.00	23.5	even	ok

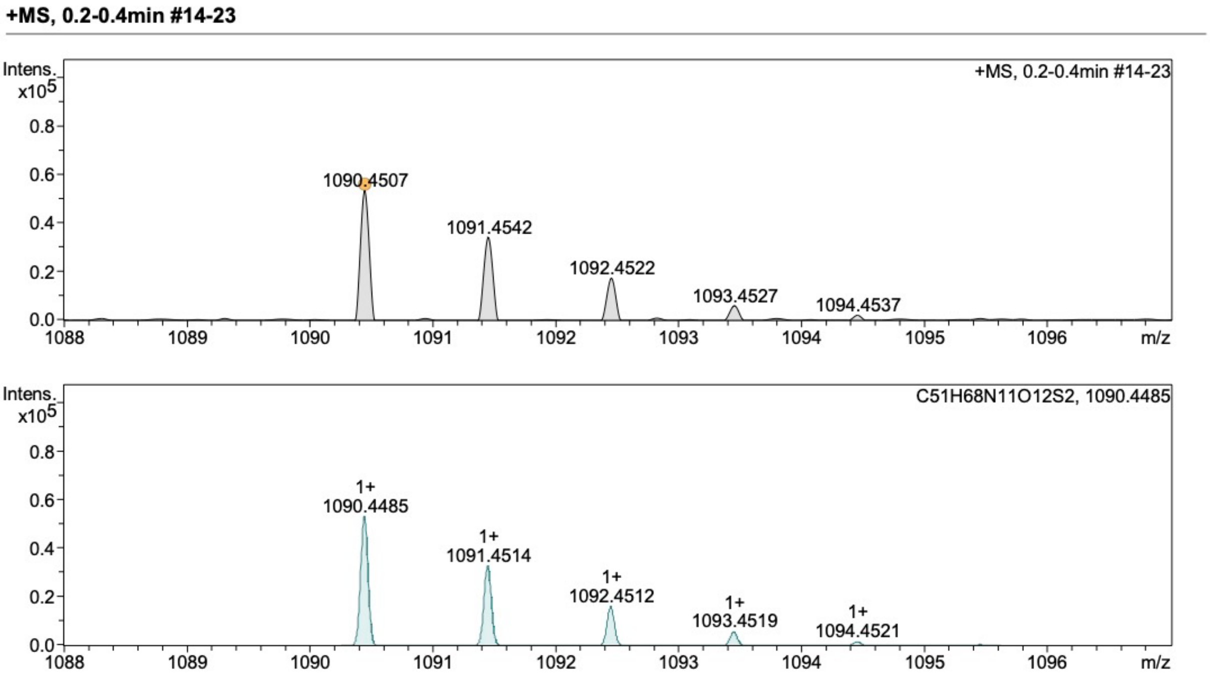

SUPPLEMENTARY FIGURES – NMR SPECTRA

^{1}H NMR spectra.

Pagoamide A isolated from *Derbesia* sp. culture extract (top) vs. synthetic pagoamide A (bottom)

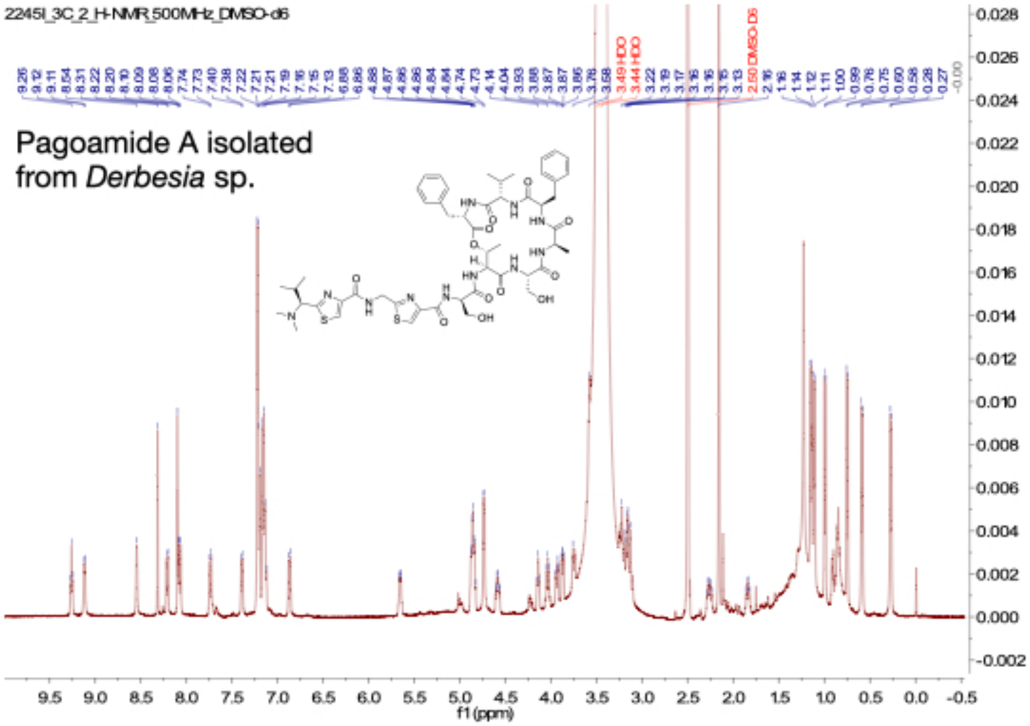

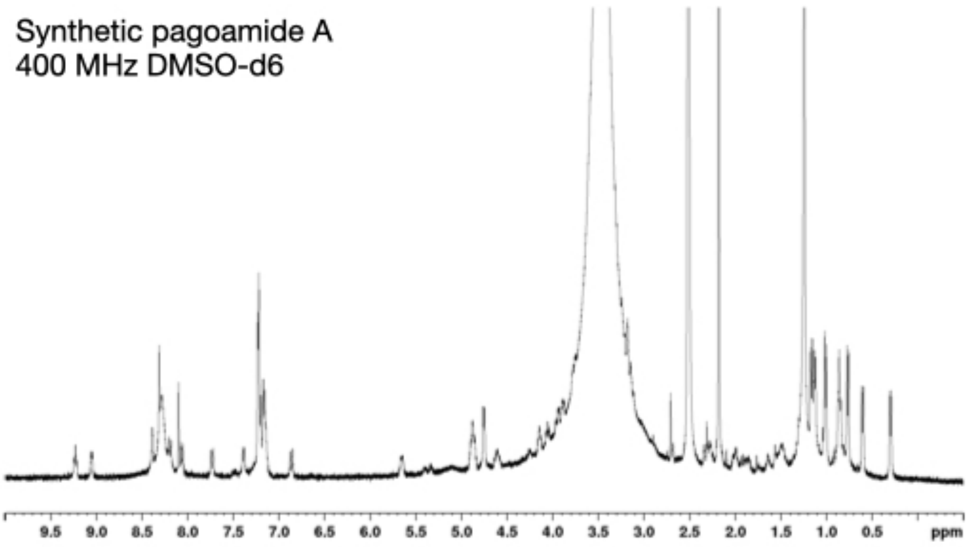

Fmoc-Gly-NH$_2$ (**5**)

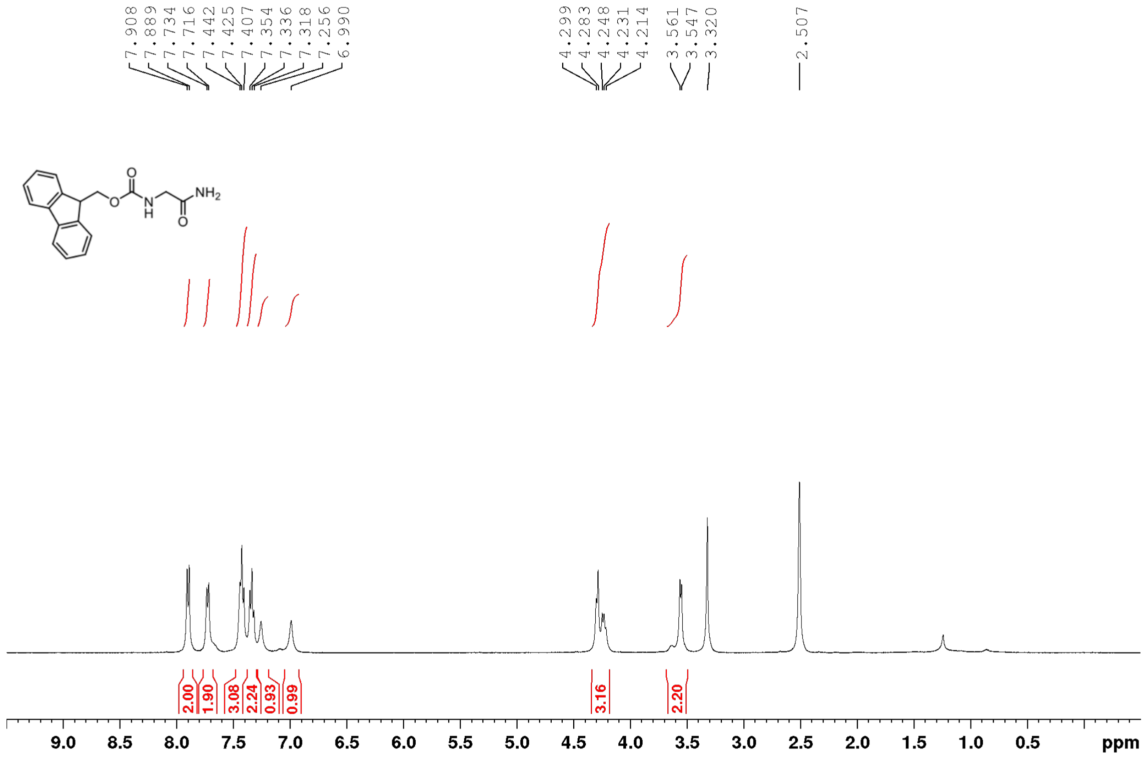

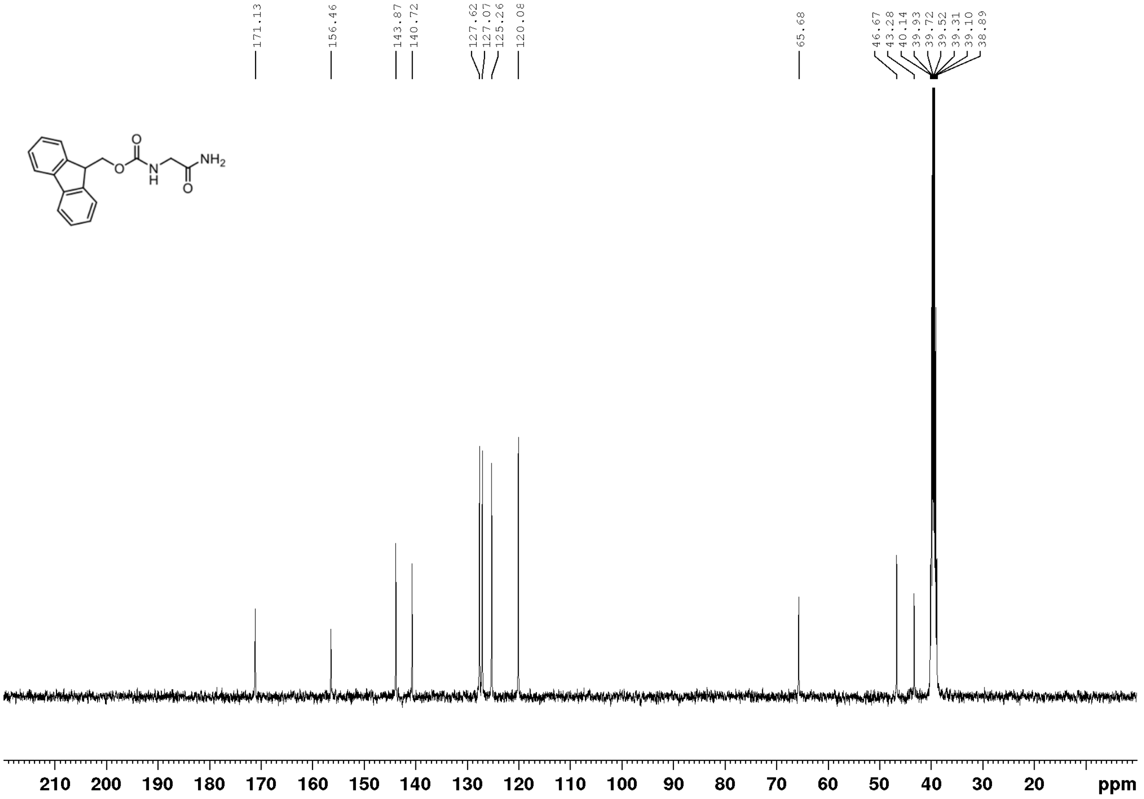

Fmoc-Gly-Thz-OH (**2**)

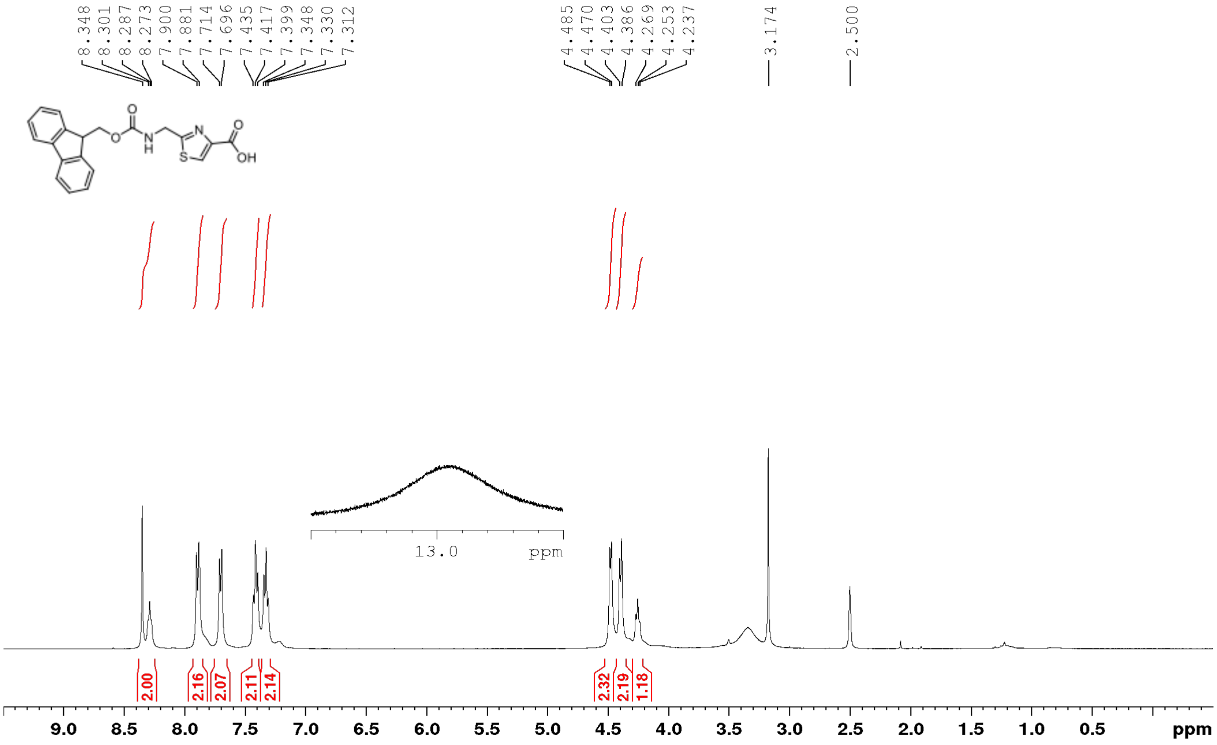

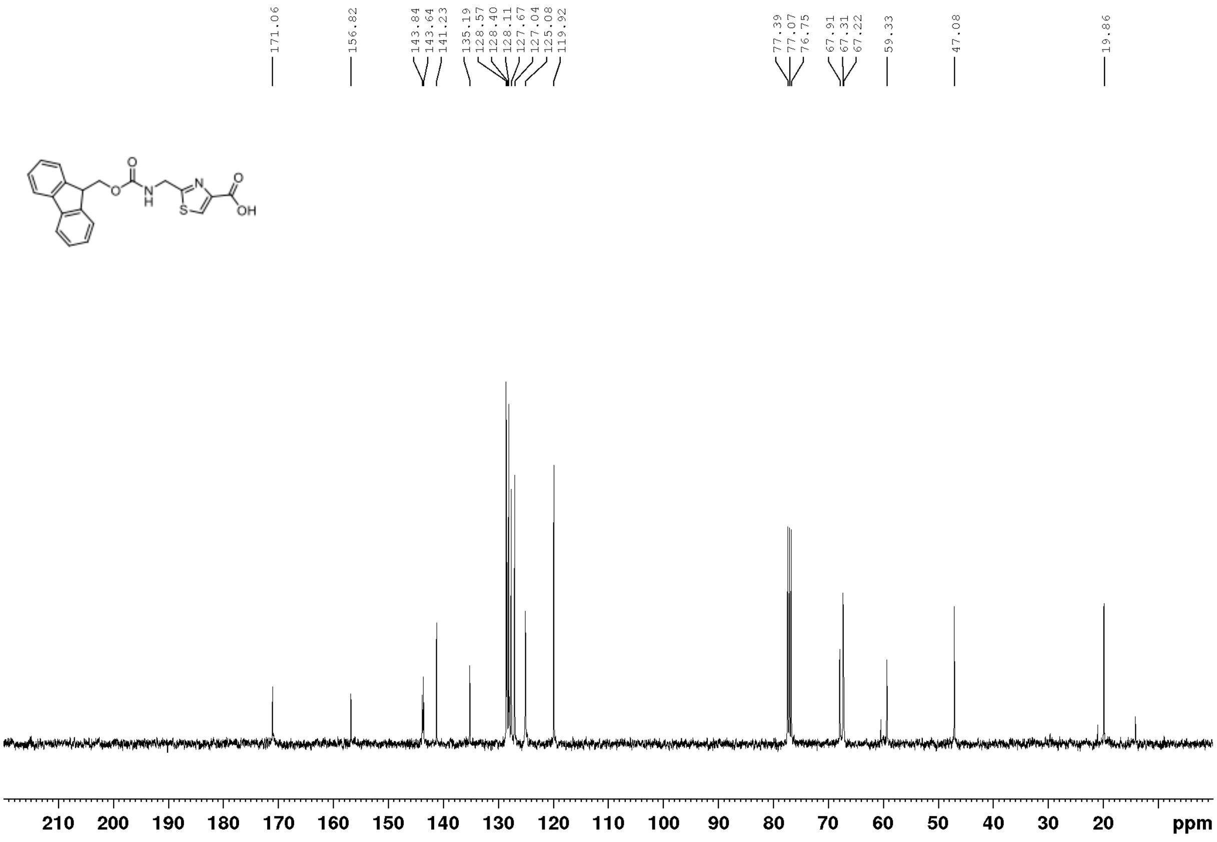

Fmoc-L-Val-NH_2 (**7**)

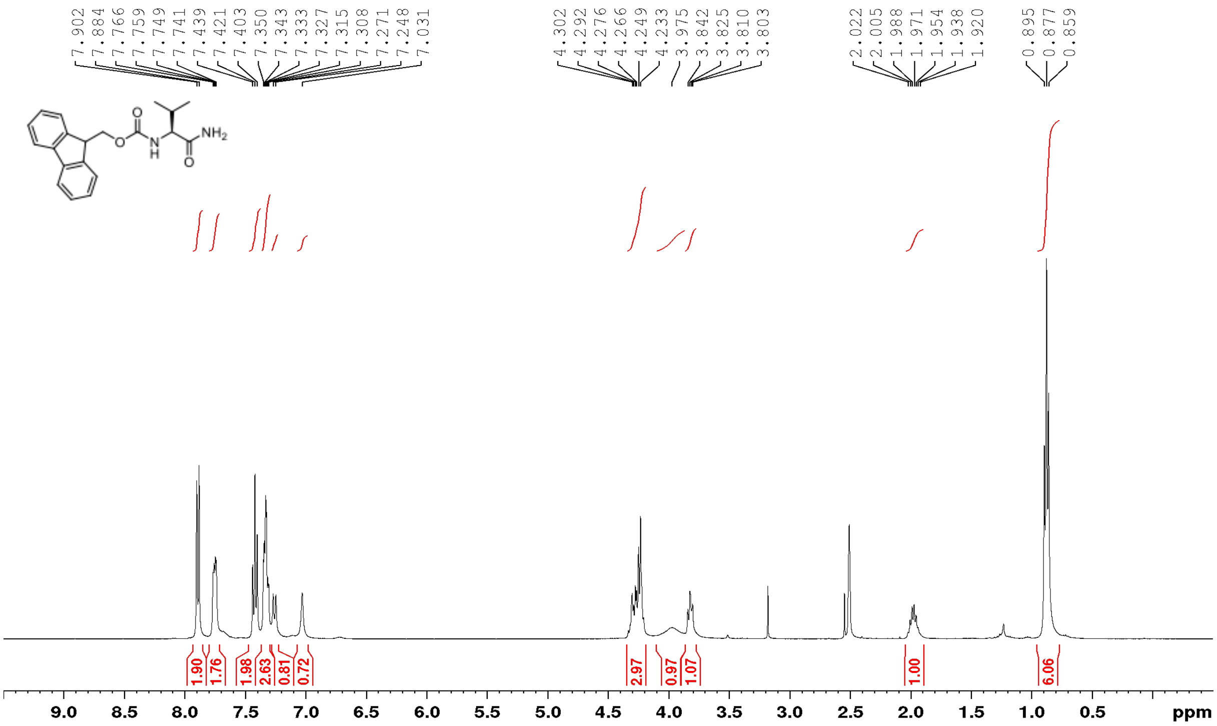

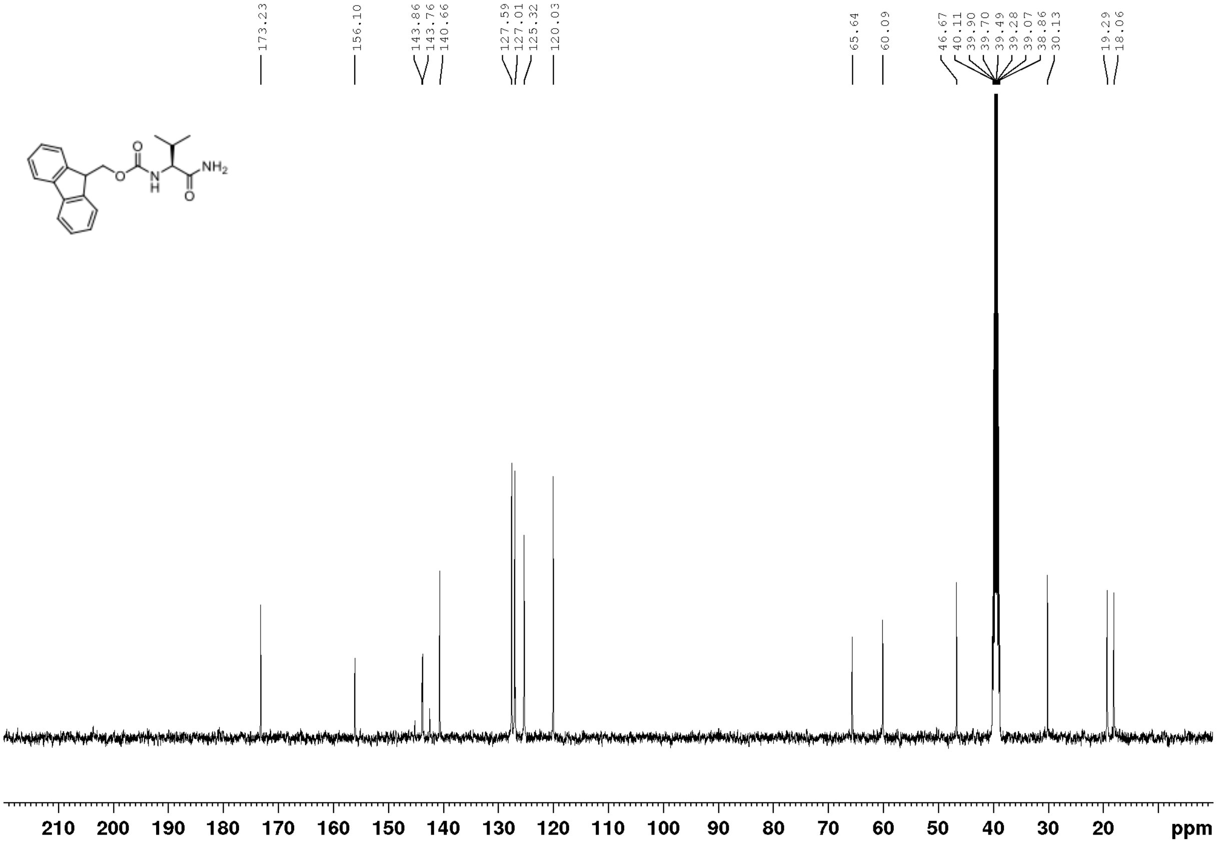

Fmoc-L-valine thioamide (**8**)

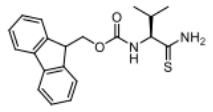

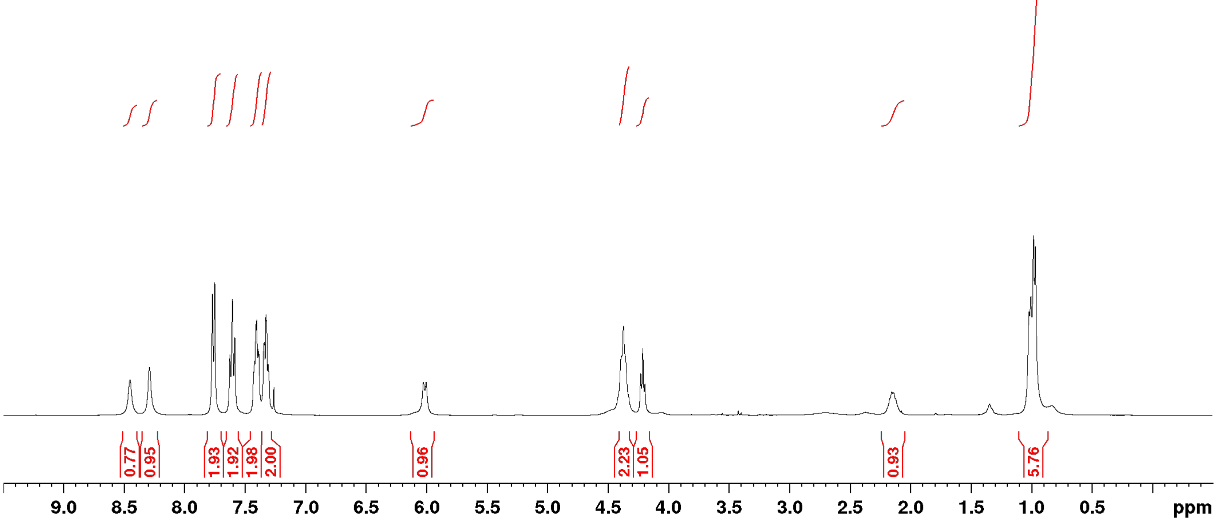

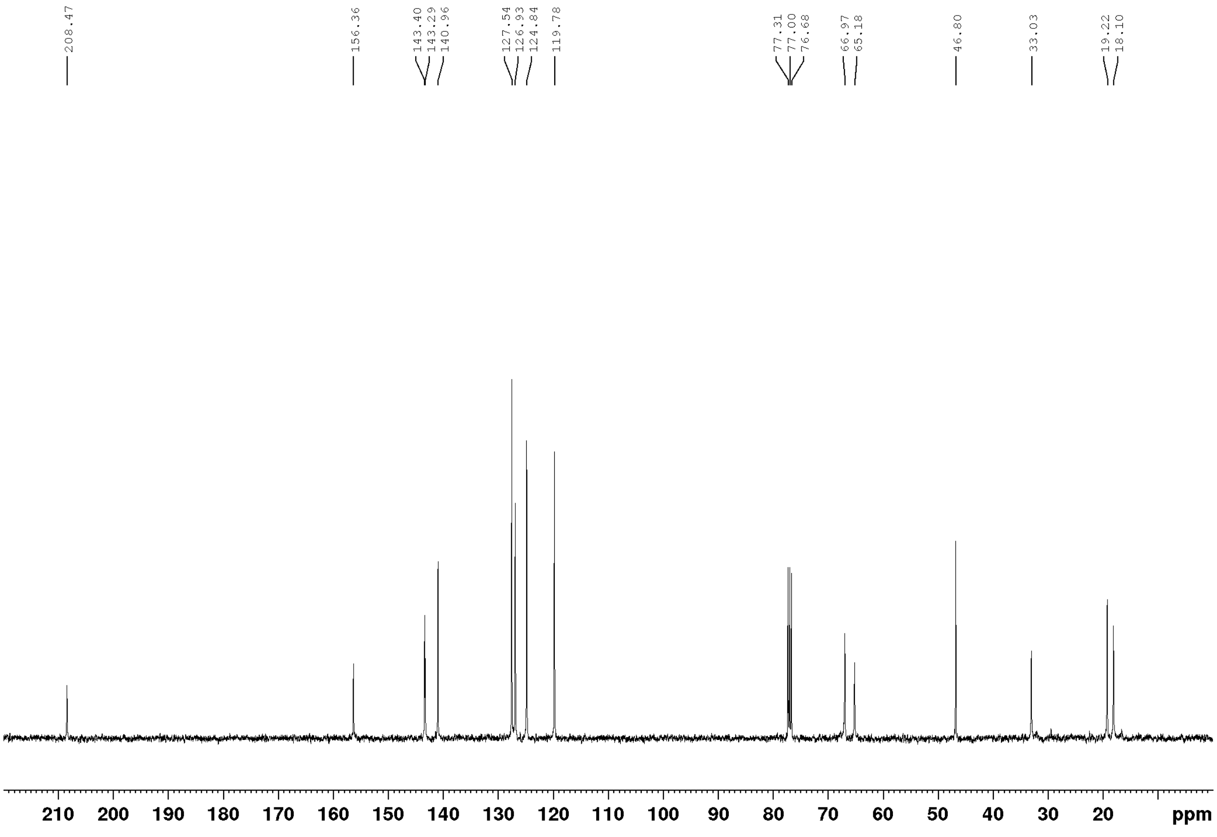

Fmoc-L-Val-Thz-OEt (**9**)

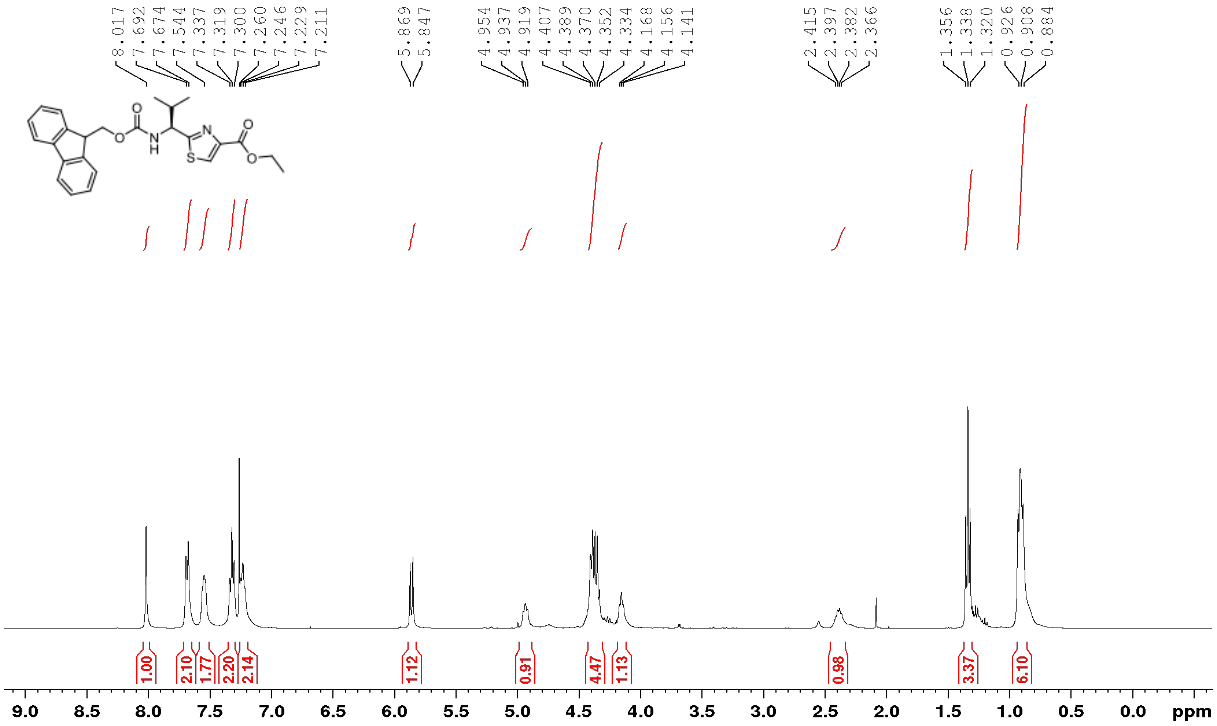

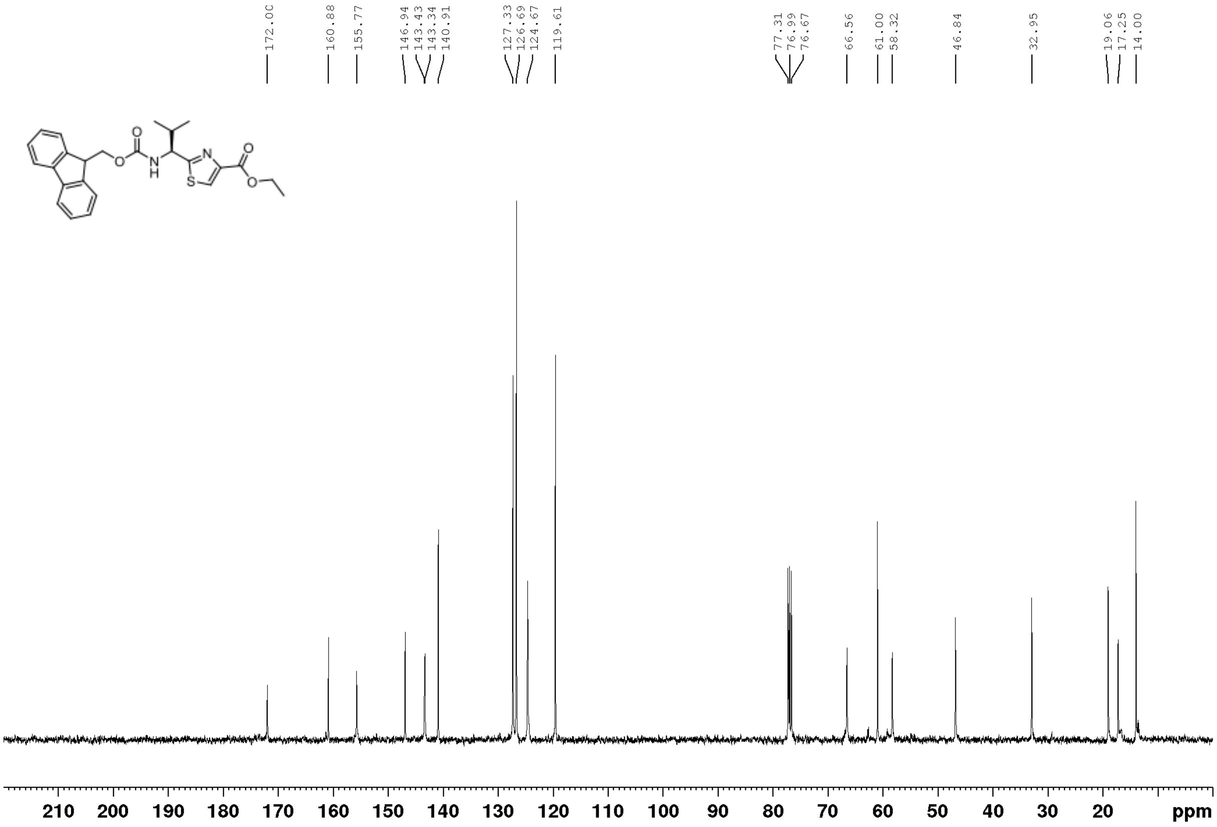

Me$_2$-L-Val-Thz-OEt (**10**)

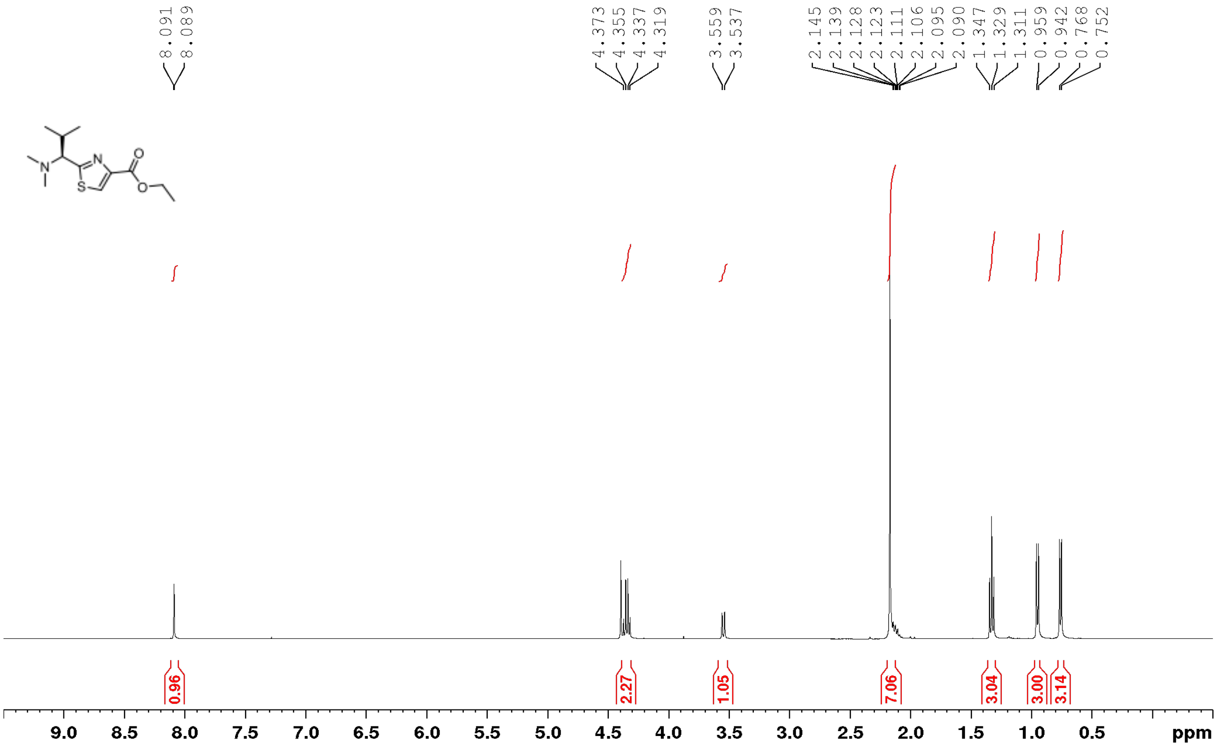

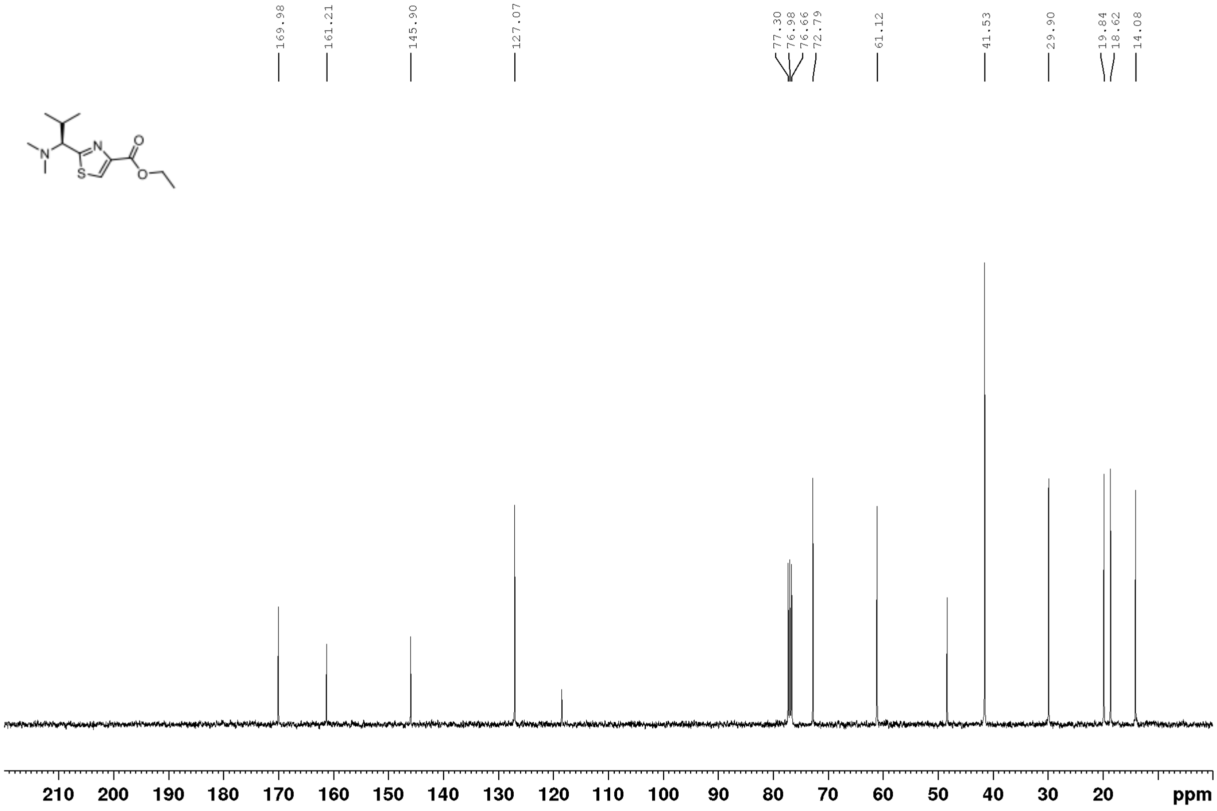

Me2-L-Val-Thz-OH (**3**)

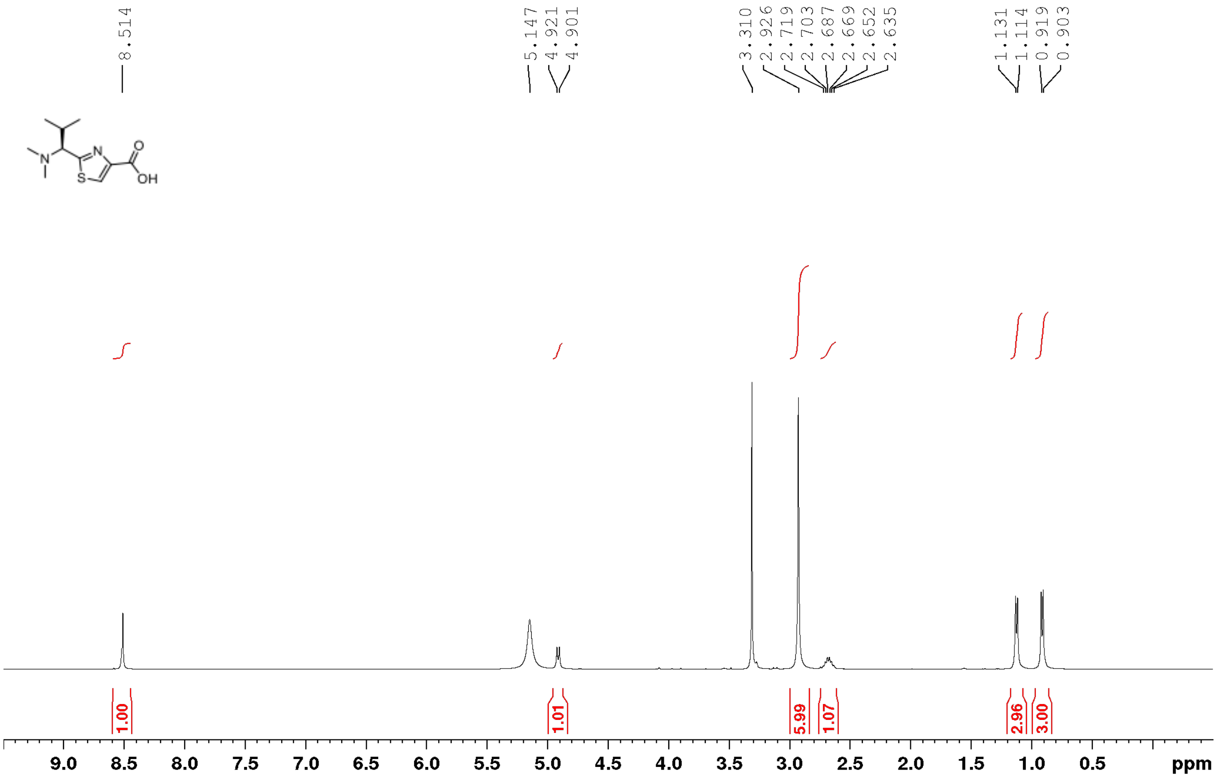

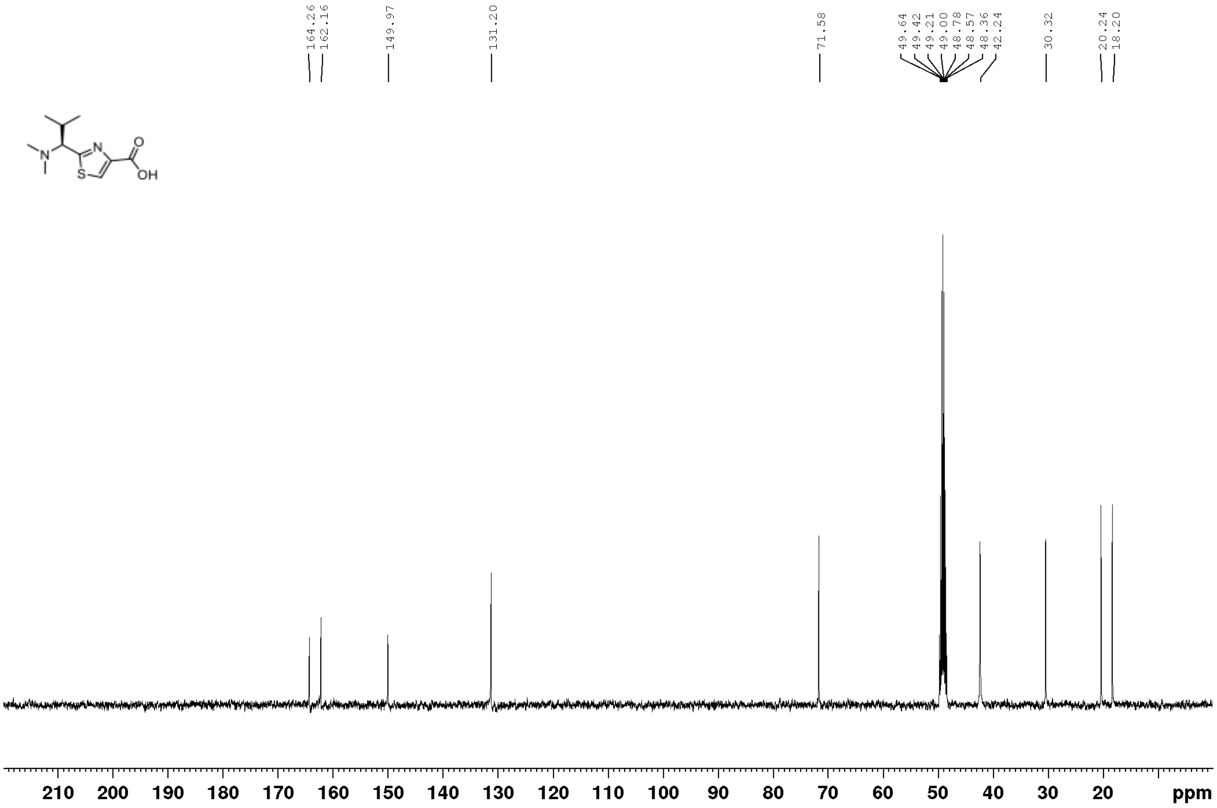

Fmoc-L-Thr-OBn (**16**)

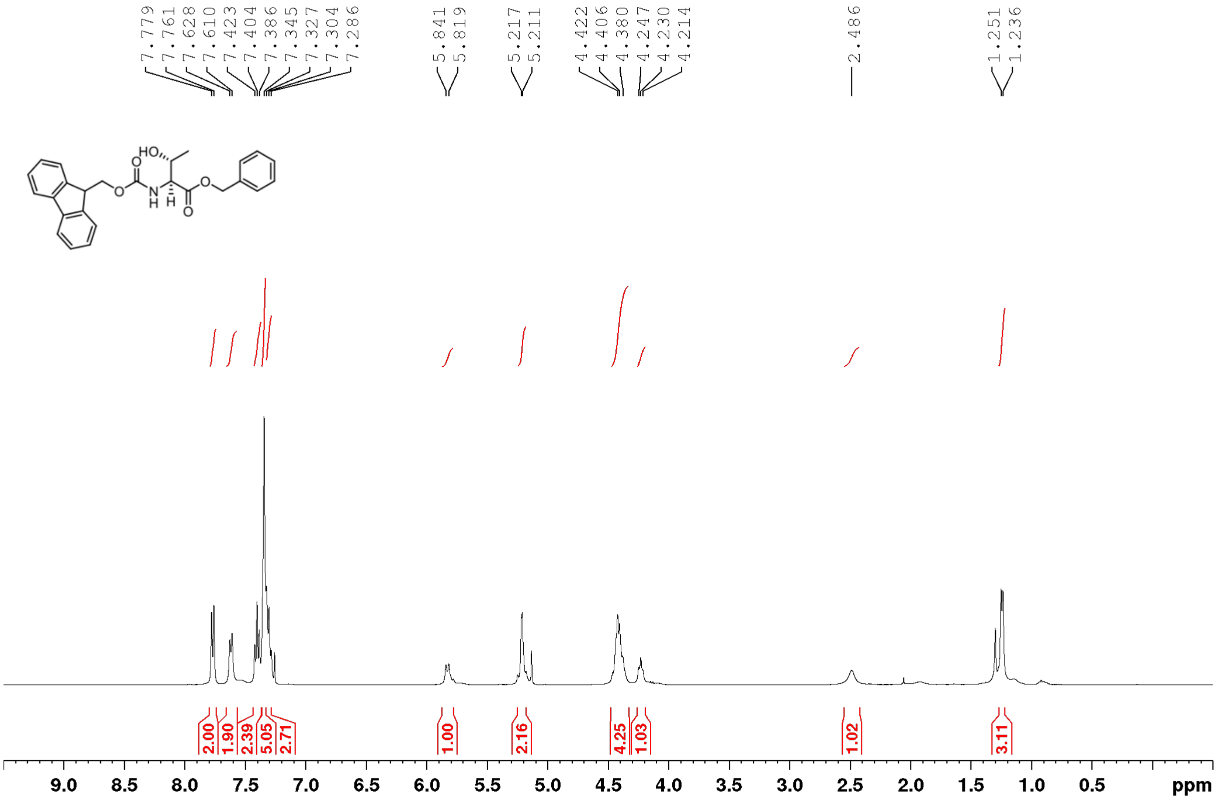

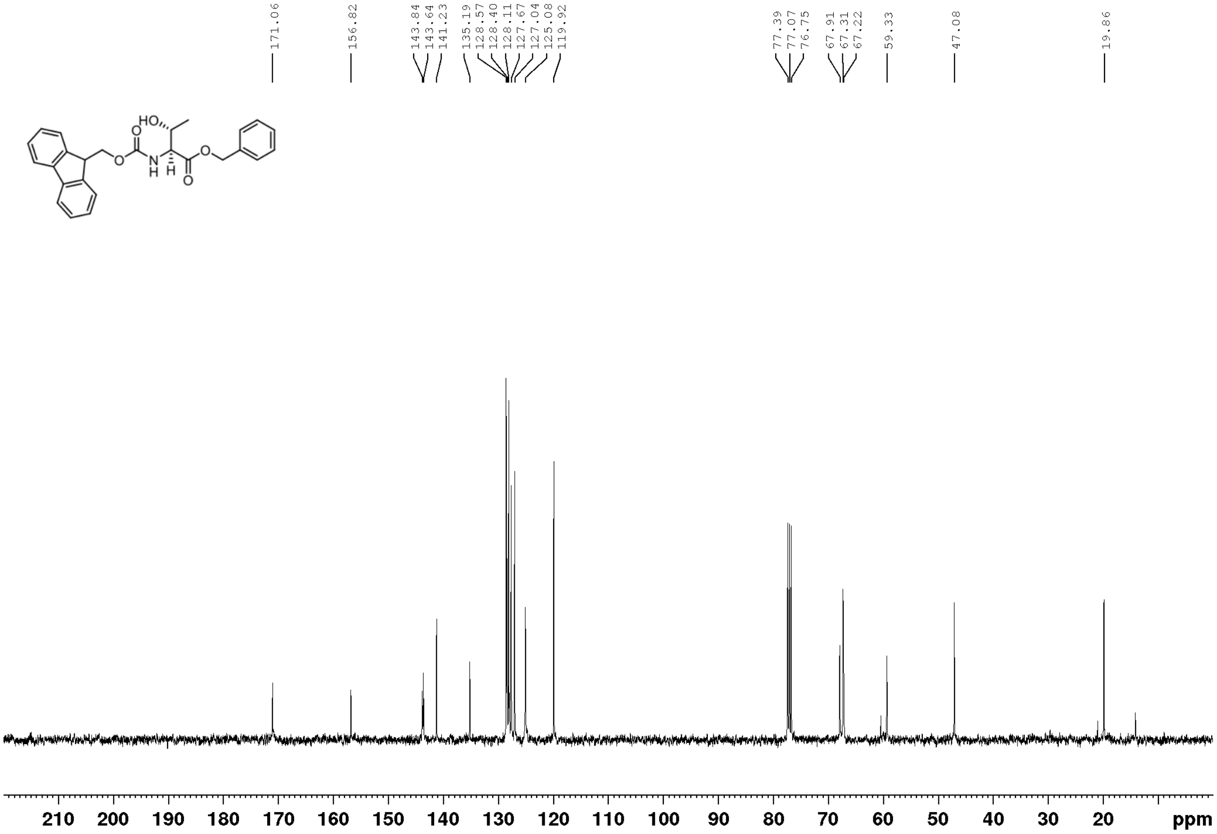

Fmoc-L-Thr(*N*-Boc-Phe)-OBn (**17**)

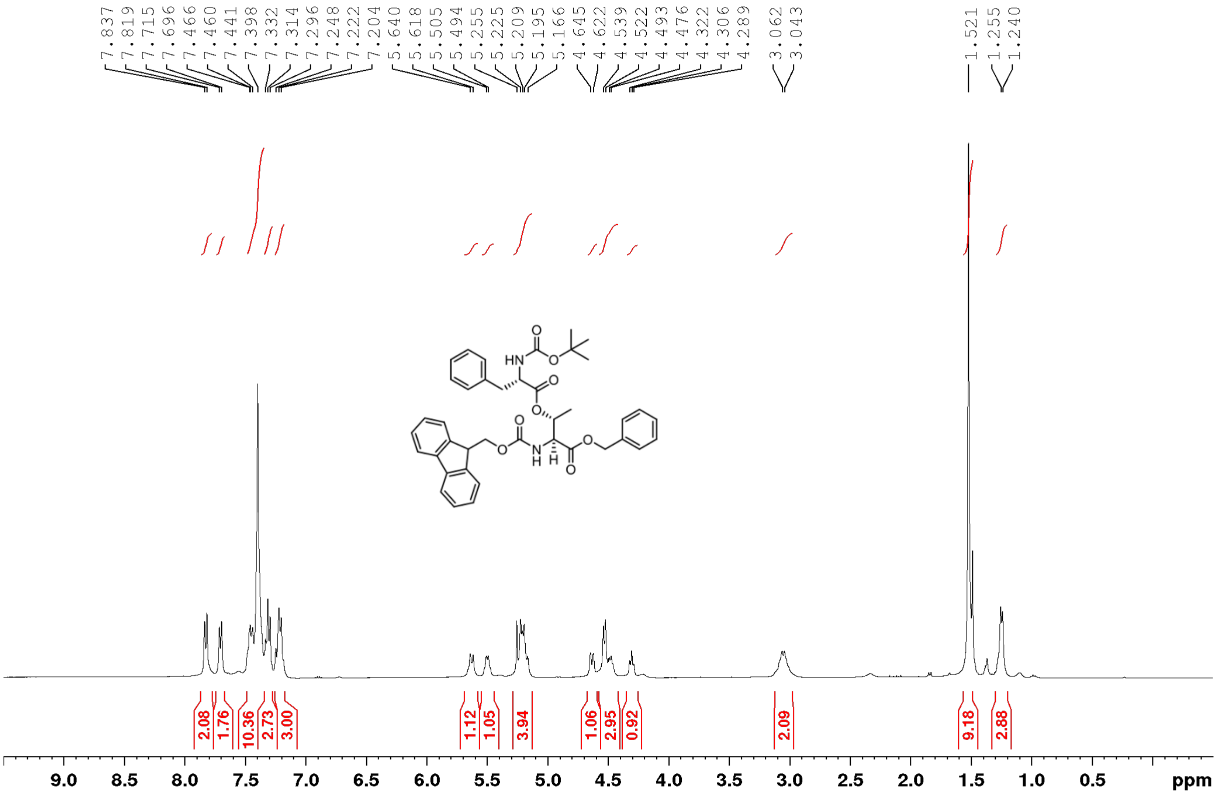

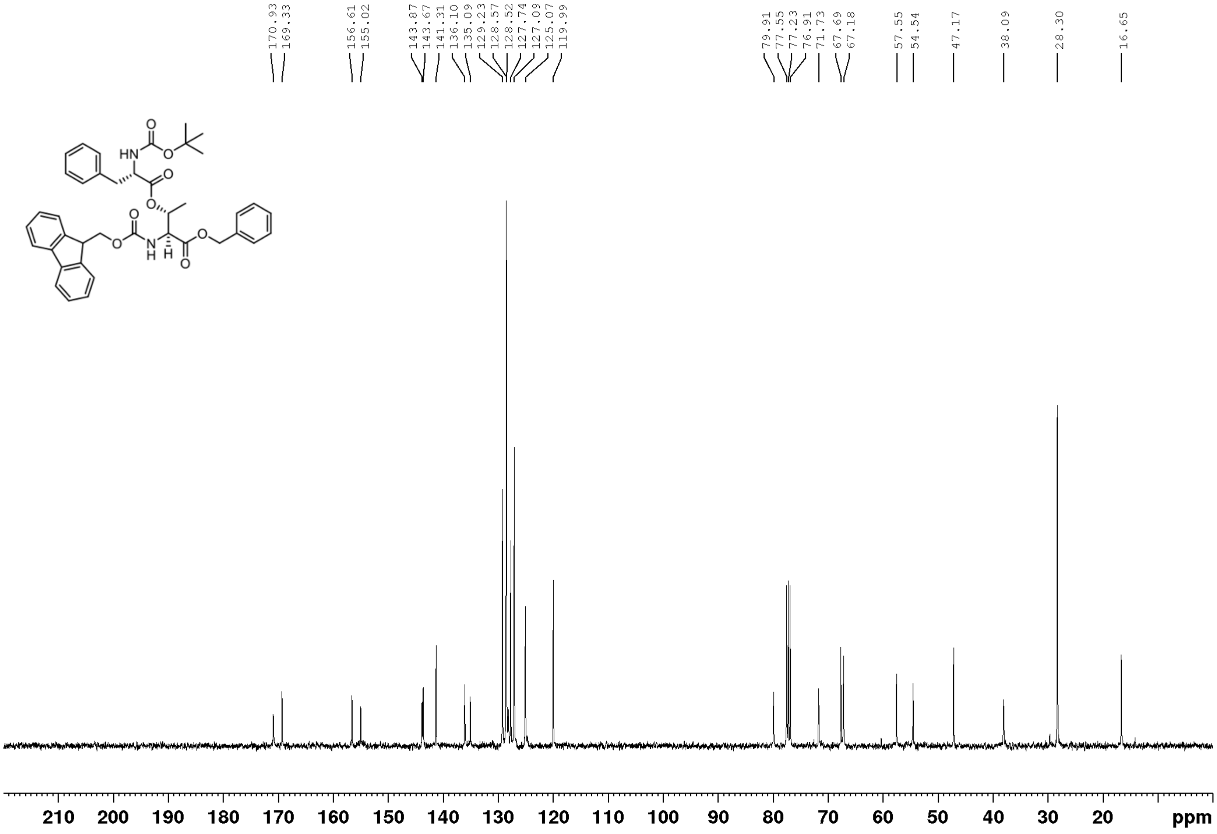

Fmoc-L-Thr(*N*-Boc-Phe)-OH (**12**)

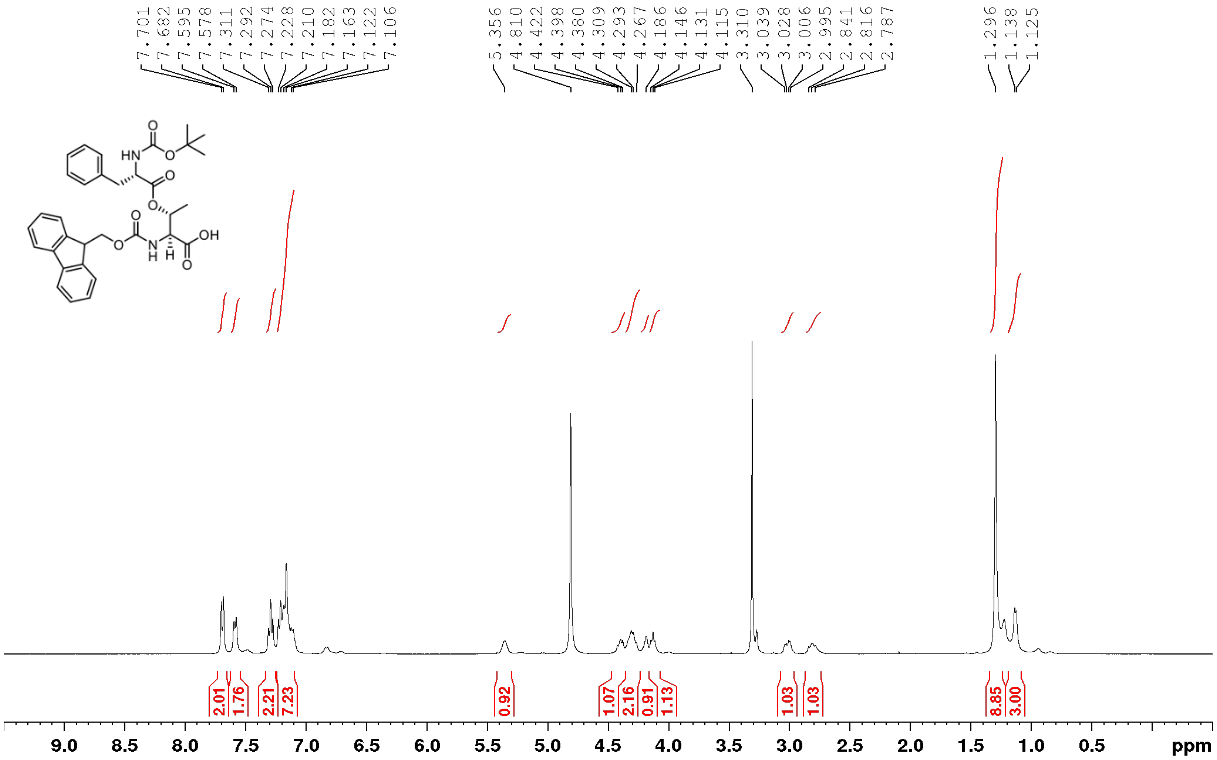

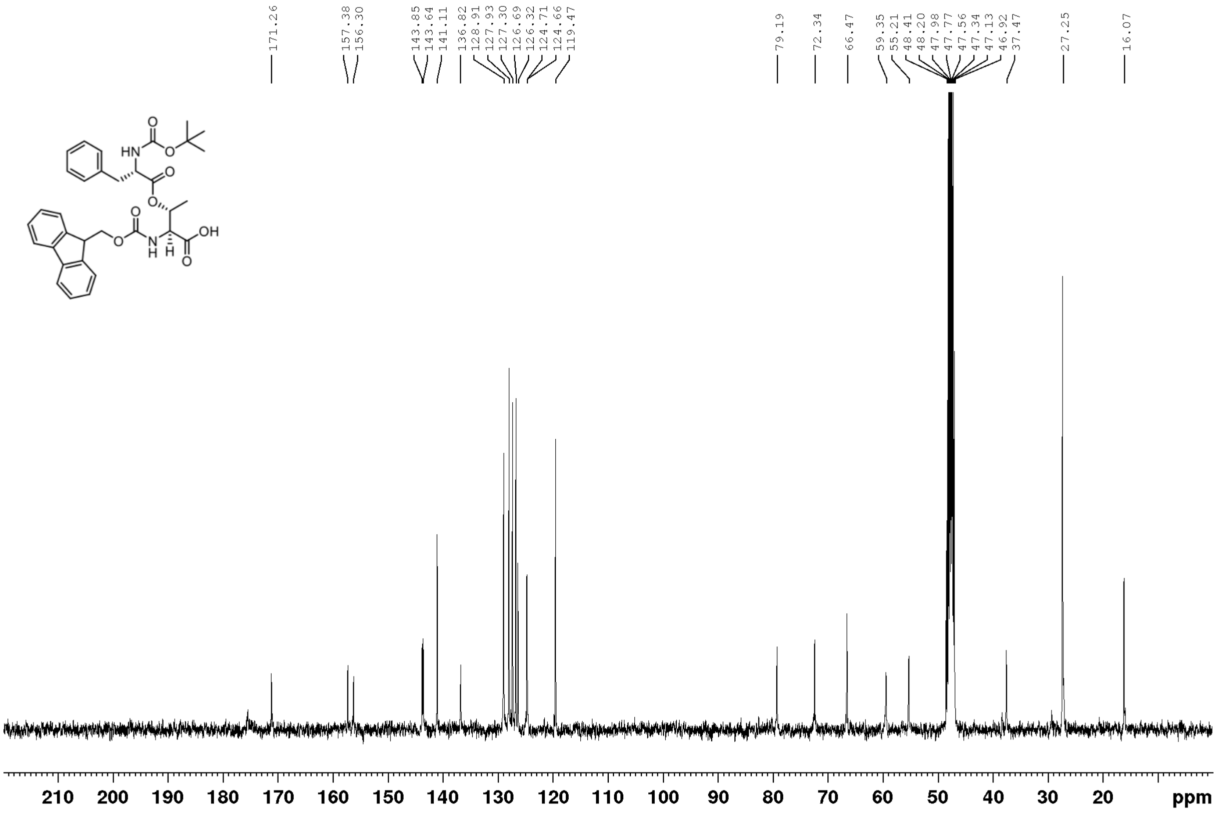

Supplement: Supplementary file 2 [file DataSheet1.pdf]
